# Supplementary material for: Nanographenes as electron-deficient cores of donor-acceptor systems
Source: Nat Commun. 2018 May 15;9:1901. doi: 10.1038/s41467-018-04321-6 (PMC5954131; doi:10.1038/s41467-018-04321-6)
Supplement: Supplementary file 1 — Supplementary Information [file 41467_2018_4321_MOESM1_ESM.pdf]

Nanographenes as Electron-Deficient Cores of Donor-Acceptor Systems

Yu-Min Liu[1‡], Hao Hou[1‡], Yan-Zhen Zhou[1], Xin-Jing Zhao[1], Chun Tang[1], Yuan-Zhi Tan[1], Klaus Müllen[2]

[1] Collaborative Innovation Center of Chemistry for Energy Materials, State Key Laboratory for Physical Chemistry of Solid Surfaces, and Department of Chemistry, College of Chemistry and Chemical Engineering, Xiamen University, Xiamen 361005

[2] Max Planck Institute for Polymer Research, Ackermannweg 10, D-55128 Mainz, Germany

Correspondence to: yuanzhi_tan@xmu.edu.cn

[‡] Y. M. Liu and H. Hou contributed equally to this work.

Supplementary Methods

The compounds **2**, **4** and **6** were characterized by ^{1}H and ^{13}C NMR spectroscopy, gel permeation chromatography (GPC) analysis, mass spectroscopy and single crystal X-ray diffraction (for **2** and **4**). The combustion of large multi-chlorinated polycyclic aromatic hydrocarbons is insufficient, which hinders elemental analysis by combustion method for compounds **2**, **4** and **6**.

Mass spectra of **2**, **4** and **6** were acquired on a Bruker microflex LRF MALDI-TOF mass spectrometer using tetracyanoquinodimethane (TCNQ) as the matrix in cation mode. As a supplement, we also tried other softer ionization sources for mass spectroscopy to avoid fragmentation and found that electrospray ionization (ESI) worked for compounds **2a-2d** and **4a-4c** (Supplementary Figures 6 and 7)as carried out on a Bruker Esquire HCT mass spectrometer (Bruker Corporation). Unfortunately, compounds **2e**, **6a** and **6b** cannot be measured by ESI under the same conditions.

All NMR spectra were acquired on a Bruker AV 500M Spectrometer or Bruker AV 600M Spectrometer with a ultra-sensitive Cryo-Probe at room temperature in the solvents indicated. Chemical shifts were expressed in ppm units relative to TMS (0.00 ppm, ^{1}H).

Ultraviolet-visible (UV-Vis) spectra in solution were recorded using a Shimadzu UV-2550 Spectrometer.

Ultraviolet-visible (UV-Vis) spectra in solid state were measured in a diffuse-reflectance mode on a Cary 5000 spectrometer. The samples for solid state absorption were prepared by mixing and grinding the crystals of NGs and $BaSO_4$ powder. Using $BaSO_4$ powder as the blank, the absorption spectra of the samples were obtained automatically by the spectrometer.

Photoluminescence spectra of **2** were measured on a Hitachi F-7000 Photoluminescence Spectrophotometer in DCM.

Crystallographic data:

2a: The single crystals were grown by slowly diffusing n-hexane into a carbon disulfide solution of **2a**.

Crystal data: monoclinic, space group $P2_1/n$ (no. 14), a = 15.8146(5) Å, b = 8.1128(3) Å, c = 26.0032(9) Å, β = 93.023(3)°, V = 3331.6(2) Å^3, Z = 2, T = 100.00(10) K, μ(CuKα) = 6.184 mm^{-1}, $Dcalc$ = 1.630 g cm^{-3}, 11425 reflections measured (6.702° ≤ 2Θ ≤ 131.81°), 5621 unique (R_{int} = 0.0571, R_{sigma} = 0.0679) which were used in all calculations. The final R_1 was 0.0552 (I > 2σ(I)) and wR_2 was 0.1571 (all data).

2c: The single crystals were grown by slow evaporation of the carbon disulfide solution of **2c**.

Crystal data: trigonal, space group R-3 (no. 148), a = 20.7857(12) Å, c = 16.9210(15) Å, V = 6331.2(9) Å^3, Z = 3, T = 100.00(10) K, μ(CuKα) = 4.034 mm^{-1}, $Dcalc$ = 1.431 g cm^{-3}, 4261 reflections measured (7.17° ≤ 2Θ ≤ 131.298°), 2354 unique (R_{int} = 0.0432, R_{sigma} = 0.0394) which were used in all calculations. The final R_1 was 0.0607 (I > 2σ(I)) and wR_2 was 0.1759 (all data).

2d: The single crystals were grown by slowly diffusing n-hexane into a carbon disulfide solution of **2d**.

Crystal data: trigonal, space group R-3 (no. 148), a = 22.3435(15) Å, c = 16.1313(14) Å, V = 6974.3(11) Å^3, Z = 3, T = 100.01(10) K, μ(MoKα) = 0.684 mm^{-1}, $Dcalc$ = 1.514 g cm^{-3}, 4226 reflections measured (6.576° ≤ 2Θ ≤ 48.898°), 2439 unique (R_{int} = 0.0479, R_{sigma} = 0.1039) which were used in all calculations. The final R_1 was 0.0947 (I > 2σ(I)) and wR_2 was 0.3118 (all data).

2e: The single crystals were grown by slow evaporation of a carbon disulfide solution of **2e**.

Crystal data: trigonal, space group R-3 (no. 148), a = 31.5274(8) Å, c = 25.7195(5) Å, V = 22139.5(12) Å^3, Z = 9, T = 100.01(10) K, μ(CuKα) = 3.459 mm^{-1}, $Dcalc$ = 1.175 g cm^{-3}, 19889 reflections measured (7.332° ≤ 2Θ ≤ 137.986°), 9120 unique (R_{int} = 0.0657, R_{sigma} = 0.0757) which were used in all calculations. The final R_1 was 0.0750 (I > 2σ(I)) and wR_2 was 0.2129 (all data).

2a⊃TTF: The single crystals were grown by slow evaporation of the carbon disulfide solution of a 1:1 mixture of **2a** and TTF. Because carbon disulfide molecules co-crystallized within the crystals, the crystals of **2a**⊃TTF easily effloresced what made the diffractions of **2a**⊃TTF relatively weak at a high angle.

Crystal data: monoclinic, space group C2/c (no. 15), a = 31.629(3) Å, b = 28.3174(17) Å, c = 46.071(3) Å, β = 106.734(7)°, V = 39516(5) Å^3, Z = 4, T = 100.01(10) K, μ(CuKα) = 5.458 mm^{-1}, $Dcalc$ = 1.383 g cm^{-3}, 53871 reflections measured (7.42° ≤ 2Θ ≤ 112.86°), 25618 unique (R_{int} = 0.1653, R_{sigma} = 0.3273) which were used in all calculations. The final R_1 was 0.1171 (I > 2σ(I)) and wR_2 was 0.3335 (all data).

4a⊃TTF: The single crystals were grown by slowly diffusing n-hexane into the carbon disulfide solution of **4a** and TTF. The crystals of **4a**⊃TTF contained carbon disulfide molecules, which made the crystals easy to effloresce when picking and mounting crystals during the measurement. As a result, the diffraction at high angle of crystals of **4a**⊃TTF disappeared.

Crystal data: triclinic, space group P-1 (no. 2), a = 13.9245(6) Å, b = 19.6503(8) Å, c = 32.4365(15) Å, α = 90.901(4)°, β = 90.656(4)°, γ = 103.718(4)°, V = 8620.0(7) Å^3, Z = 2, T = 99.9(4) K, μ(CuKα) = 5.728 mm^{-1},$Dcalc$ = 1.417 g cm^{-3}, 29448 reflections measured (7.042° ≤ 2Θ ≤ 89.798°), 13553 unique (R_{int} = 0.0663, R_{sigma} = 0.0941) which were used in all calculations. The final R_1 was 0.1262 (I > 2σ(I)) and wR_2 was 0.3240 (all data).

4c⊃TTF: The single crystals were grown by slowly diffusing n-hexane into the carbon disulfide solution of **4c** and TTF.

Crystal data: triclinic, space group P-1 (no. 2), a = 21.5223(6) Å, b = 23.1734(8) Å, c = 26.4204(6) Å, α = 108.025(3)°, β = 98.092(2)°, γ = 115.886(3)°, V = 10671.0(6) Å^3, Z = 2, T = 100.01(10) K, μ(CuKα) = 4.953 mm^{-1}, $Dcalc$ = 1.369 g cm^{-3}, 66796 reflections measured (6.938° ≤ 2Θ ≤ 126.108°), 33436 unique (R_{int} = 0.0810, R_{sigma} = 0.0816) which were used in all calculations. The final R_1 was 0.0968 (I > 2σ(I)) and wR_2 was 0.2866 (all data).

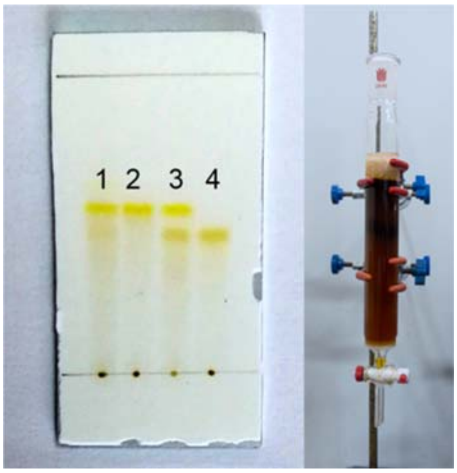

Supplementary Figure 1 Thin layer chromatography (TLC) analysis of crude products (spot 1), major component collected as **2a** (spot 2), subsequent eluent (spot 3), isolatable byproduct (spot 4) and the photo of silica column after separation. The byproduct (spot 4) was obtained by additional chromatographic separation.

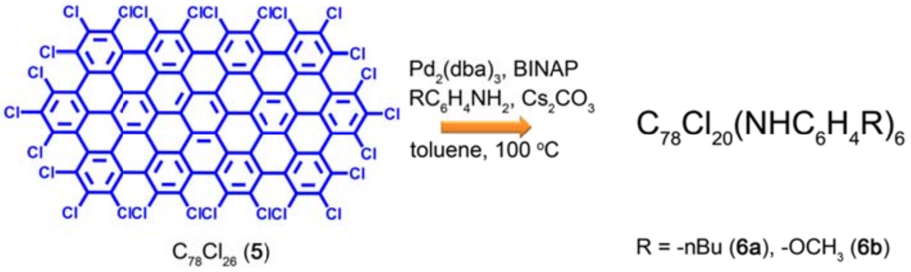

Supplementary Figure 2 Synthetic scheme for **6** from **5**.

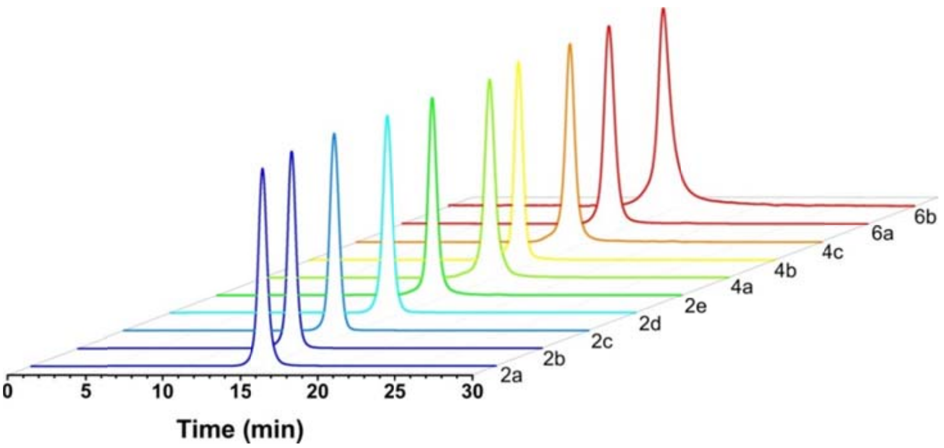

Supplementary Figure 3 GPC analysis of compounds **2**, **4** and **6**. All the chromatographs were acquired using a JAIGEL-2H column eluted by $CHCl_3$ at a flow rate of 6 ml/min, monitored at 330 nm by a UV-Vis detector. All the compounds showed a sharp chromatographic peak, confirming their purity. The retention times of **2a-2e**, **4a-4c** and **6a-6b** is 14.95, 13.80, 13.50, 14.00, 13.95, 14.55, 13.40, 13.90, 13.25 and 13.80 min, respectively.

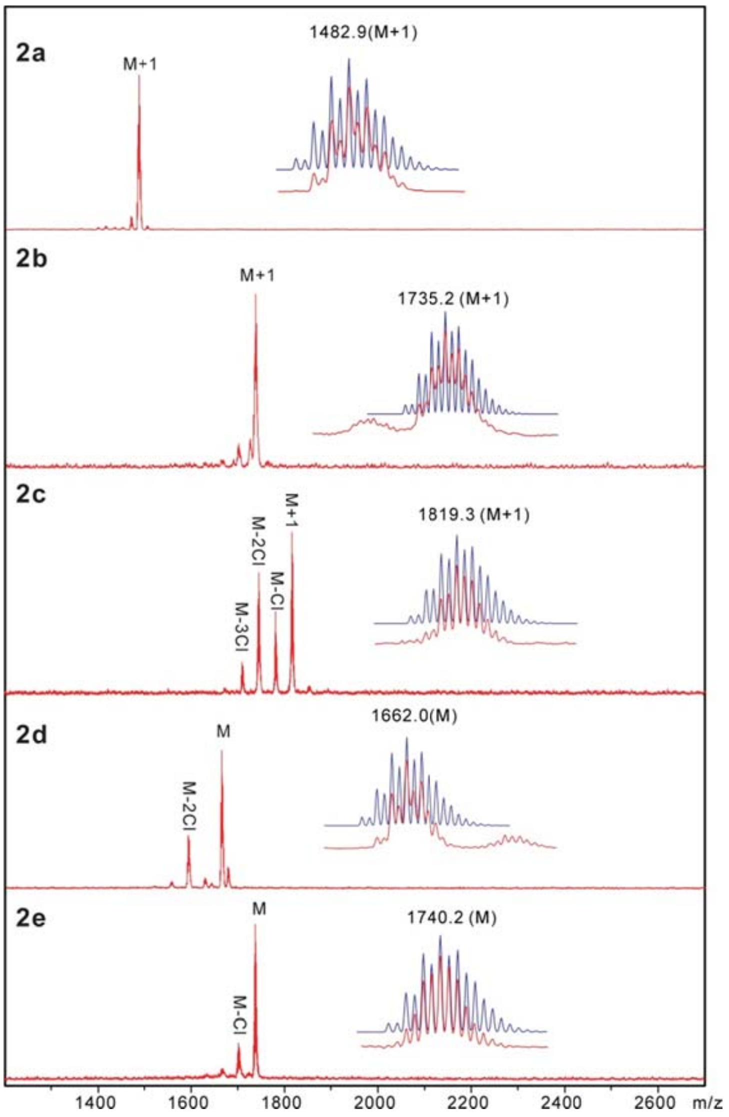

Supplementary Figure 4 Mass spectra of **2** acquired on a Bruker microflex LRF MALDI-TOF mass spectrometer using TCNQ as the matrix in cation mode. The experimental and calculated spectra were represented in red and blue, respectively.

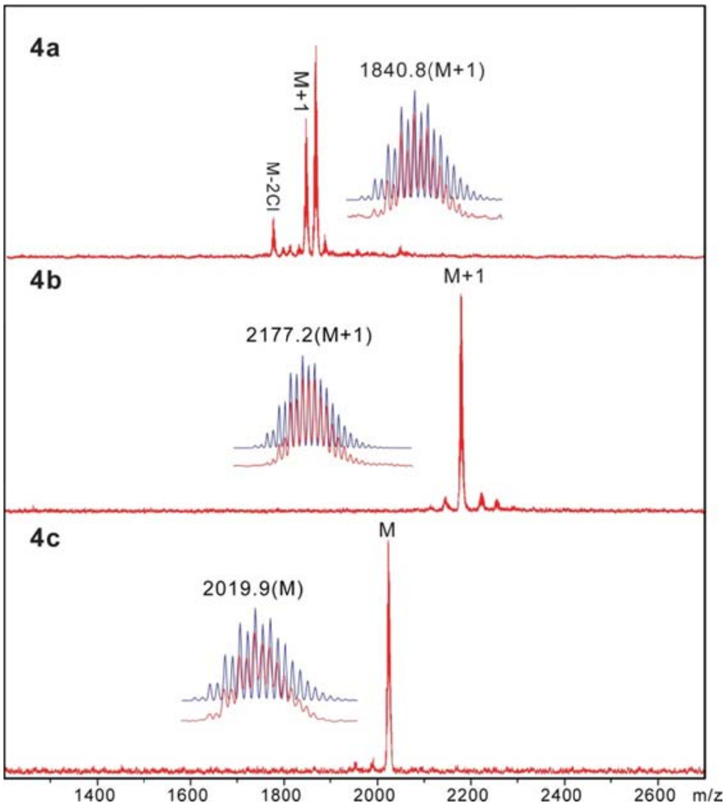

Supplementary Figure 5 Mass spectra of **4** acquired on a Bruker microflex LRF MALDI-TOF mass spectrometer using TCNQ as matrix in cation mode. The experimental and calculated spectra were represented in red and blue, respectively.

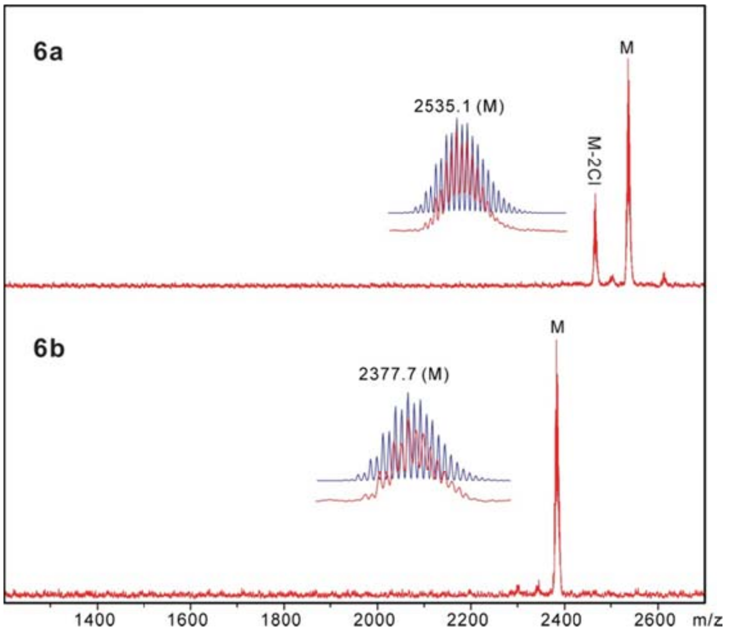

Supplementary Figure 6 Mass spectra of **6** acquired on a Bruker microflex LRF MALDI-TOF mass spectrometer using TCNQ as matrix in cation mode. The experimental and calculated spectra represented in red and blue, respectively, fit well. The formula of **6** can be assigned as $C_{78}Cl_{20}(NHC_6H_4R)_6$ (R= $-C_4H_9$ for **6a** and $-OCH_3$ for **6b**).

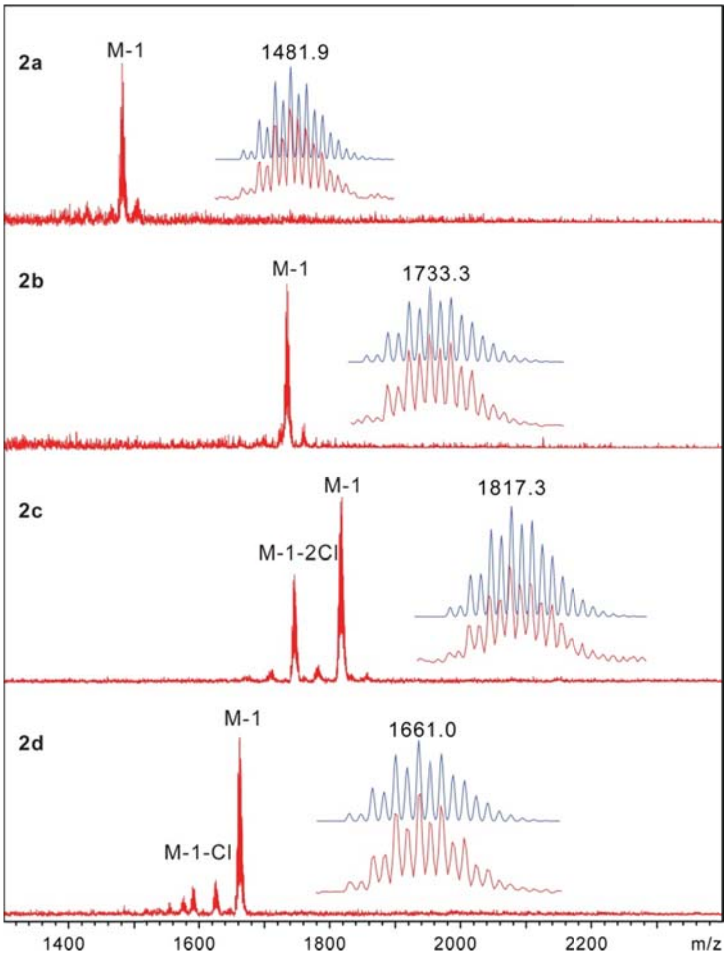

Supplementary Figure 7 Mass spectra of **2a-2d** acquired on a Bruker Esquire HCT mass spectrometer using ESI in anion mode. The experimental and calculated spectra were represented in red and blue, respectively.

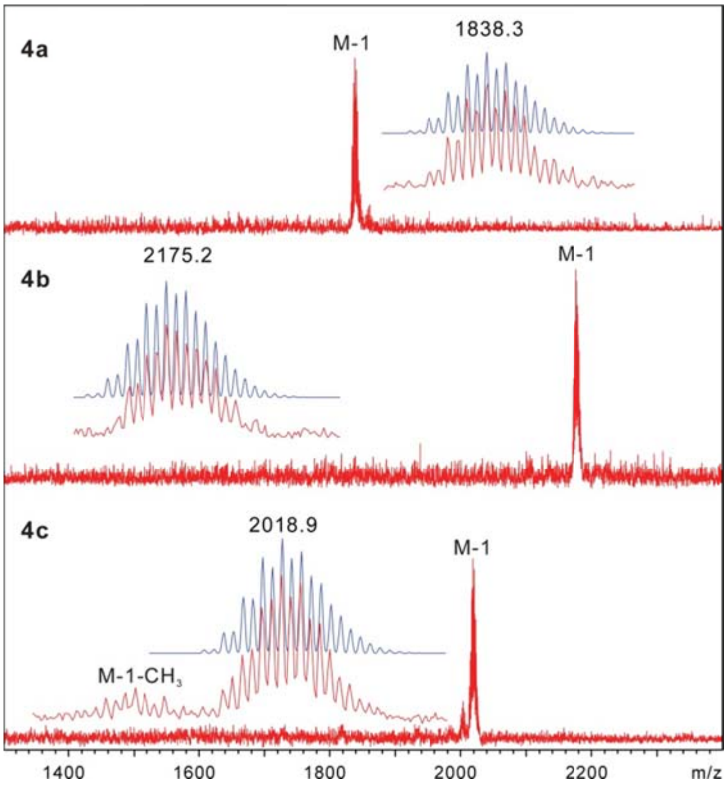

Supplementary Figure 8 Mass spectra of **4a-4c** acquired on a Bruker Esquire HCT mass spectrometer using ESI in anion mode. The experimental and calculated spectra were represented in red and blue, respectively.

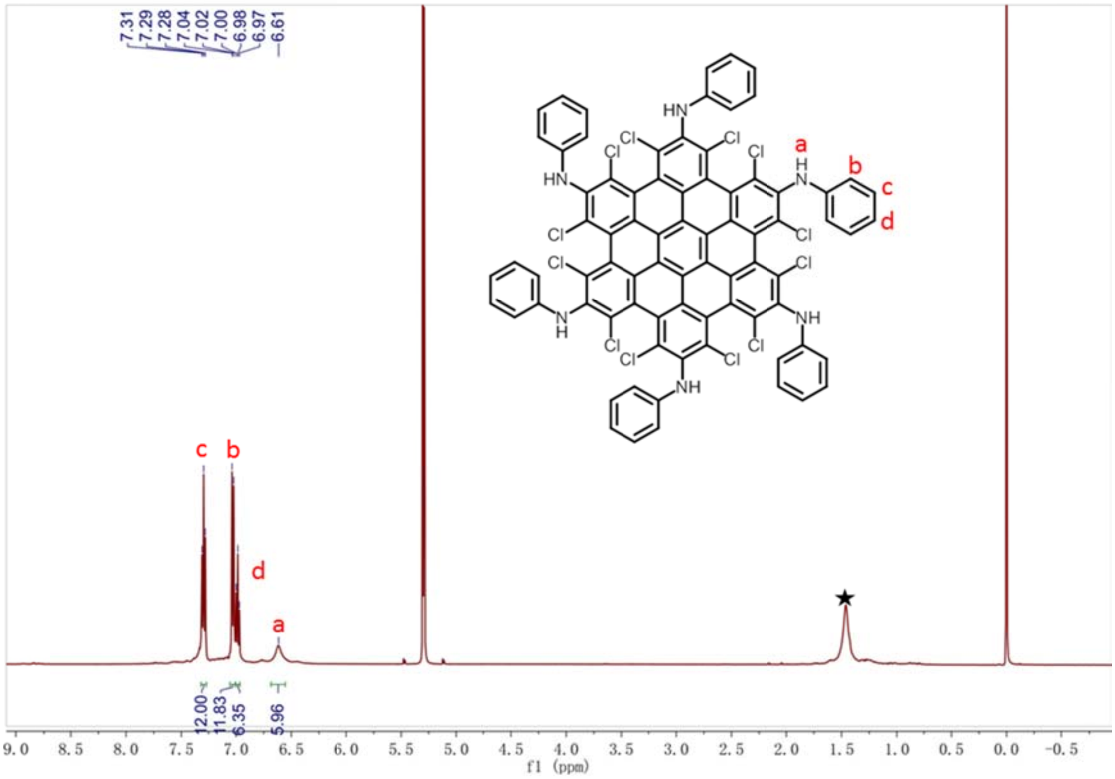

Supplementary Figure 9 ^{1}H NMR spectrum of **2a** in CD_2Cl_2. The peak at 1.50 ppm was assigned to the signal of water, marked by asterisk. ^{1}H NMR (500 MHz, CD_2Cl_2) δ 7.29 (t, J = 7.9 Hz, 12H), 7.03 (d, J = 7.8 Hz, 12H), 6.98 (t, J = 7.4 Hz, 6H), 6.61 (s, 6H) ppm.

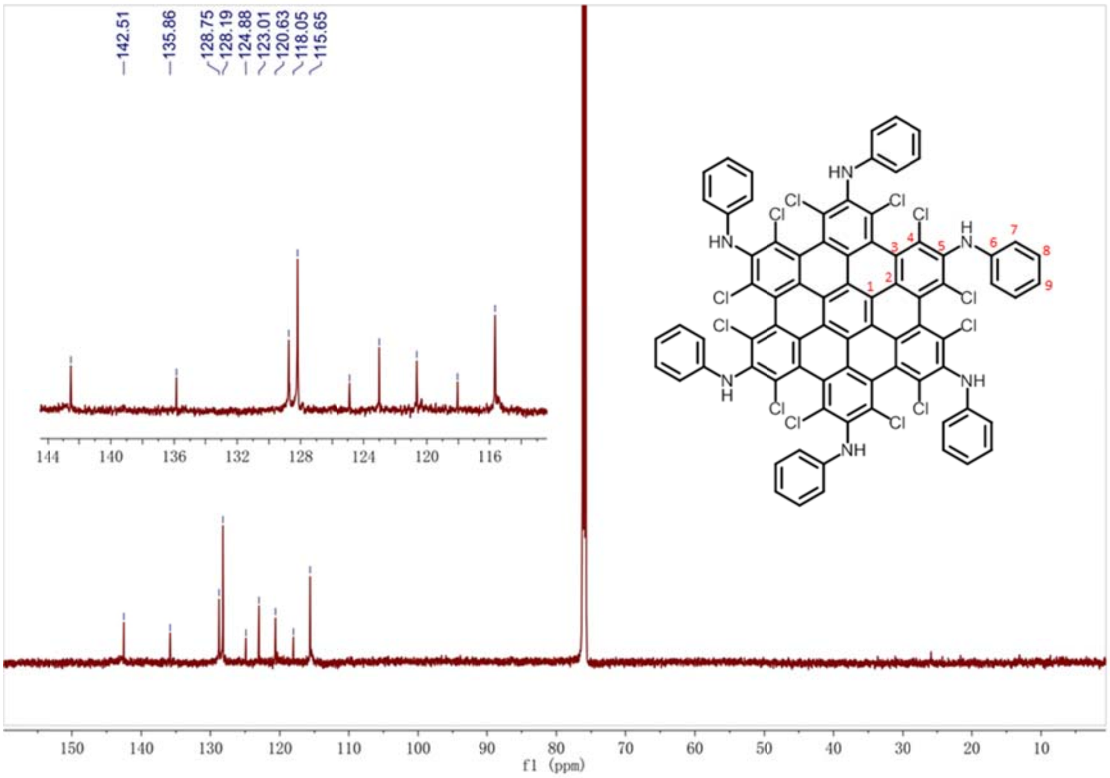

Supplementary Figure 10 ^{13}C NMR spectrum of **2a** in $CDCl_3$. ^{13}C NMR (151 MHz, $CDCl_3$) δ 142.51, 135.86, 128.75, 128.19, 124.88, 123.01, 120.63, 118.05, 115.65 ppm.

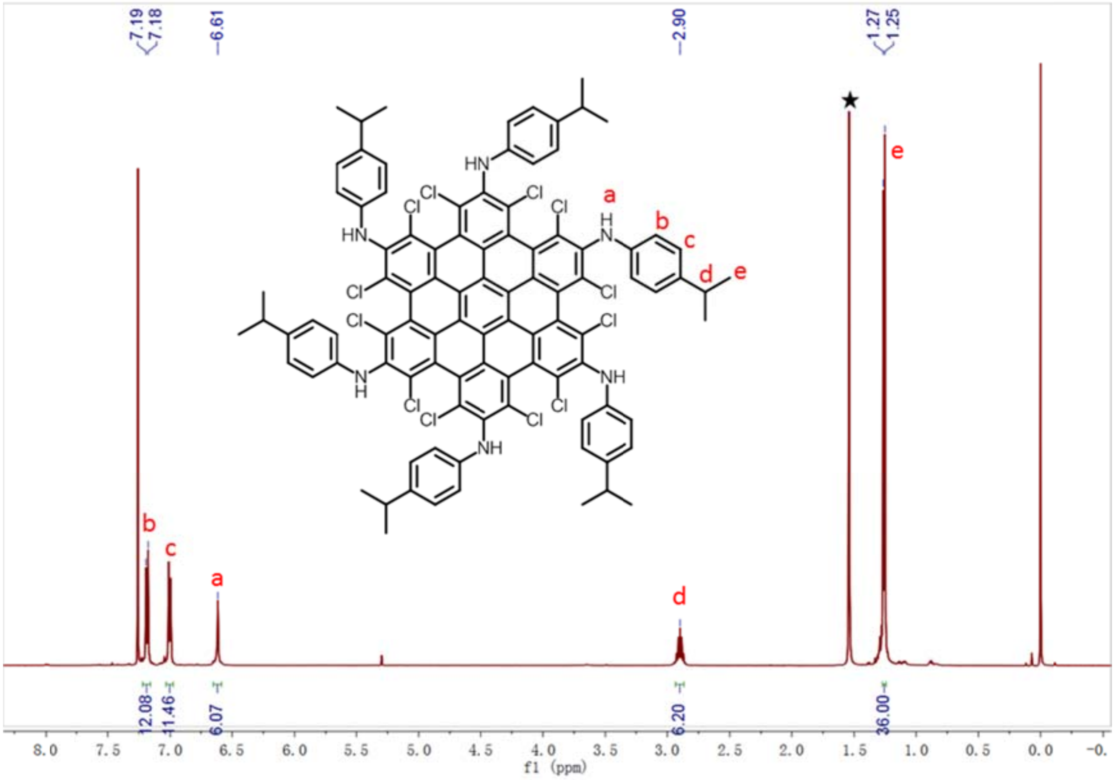

Supplementary Figure 11 ^{1}H NMR spectrum of **2b** in $CDCl_3$. The peaks at 1.52 ppm was assigned to the signal of water, marked by asterisk. ^{1}H NMR (500 MHz, $CDCl_3$): δ 7.18 (d, J = 7.5 Hz, 12H), 7.00 (d, J = 8.4 Hz, 12H), 6.61 (s 6H), 2.90 (m, 6H), 1.26 (d, J = 6.3 Hz, 36H) ppm.

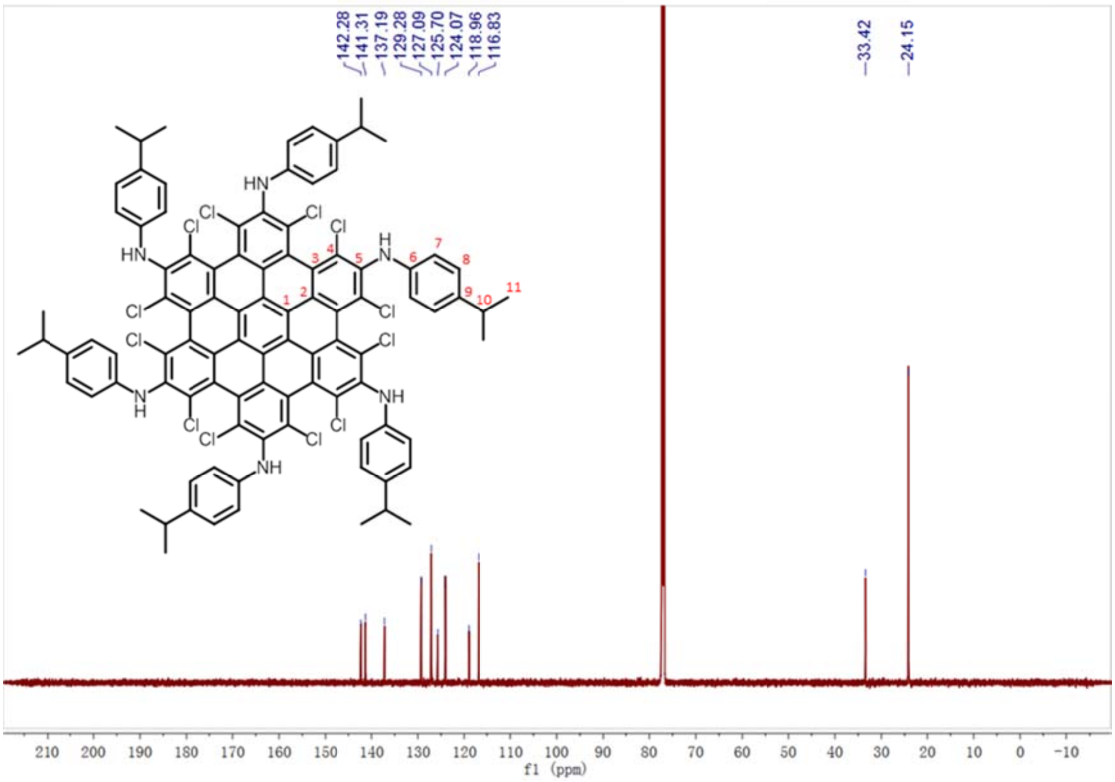

Supplementary Figure 12 ^{13}C NMR spectrum of **2b** in $CDCl_3$. ^{13}C NMR (151 MHz, $CDCl_3$) δ 142.28, 141.31, 137.19, 129.28, 127.09, 125.70, 124.07, 118.96, 116.83, 33.42, 24.15 ppm.

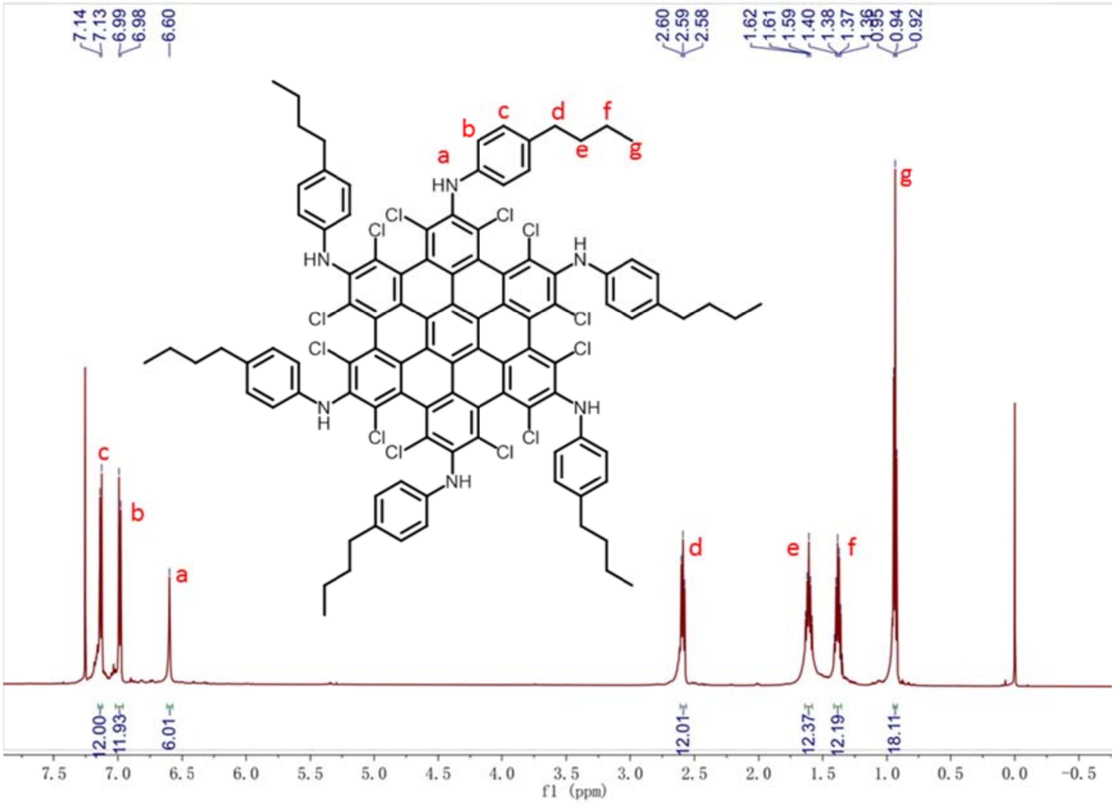

Supplementary Figure 13 ^{1}H NMR spectrum of **2c** in $CDCl_3$. ^{1}H NMR (600 MHz, $CDCl_3$) δ 7.13 (d, J = 8.4 Hz, 12H), 6.98 (d, J = 8.4 Hz, 12H), 6.60 (s, 6H), 2.59 (t, J = 6.8 Hz, 12H), 1.64 – 1.58 (m, 12H), 1.42 – 1.34 (m, 12H), 0.94 (t, J = 7.0 Hz, 18H) ppm.

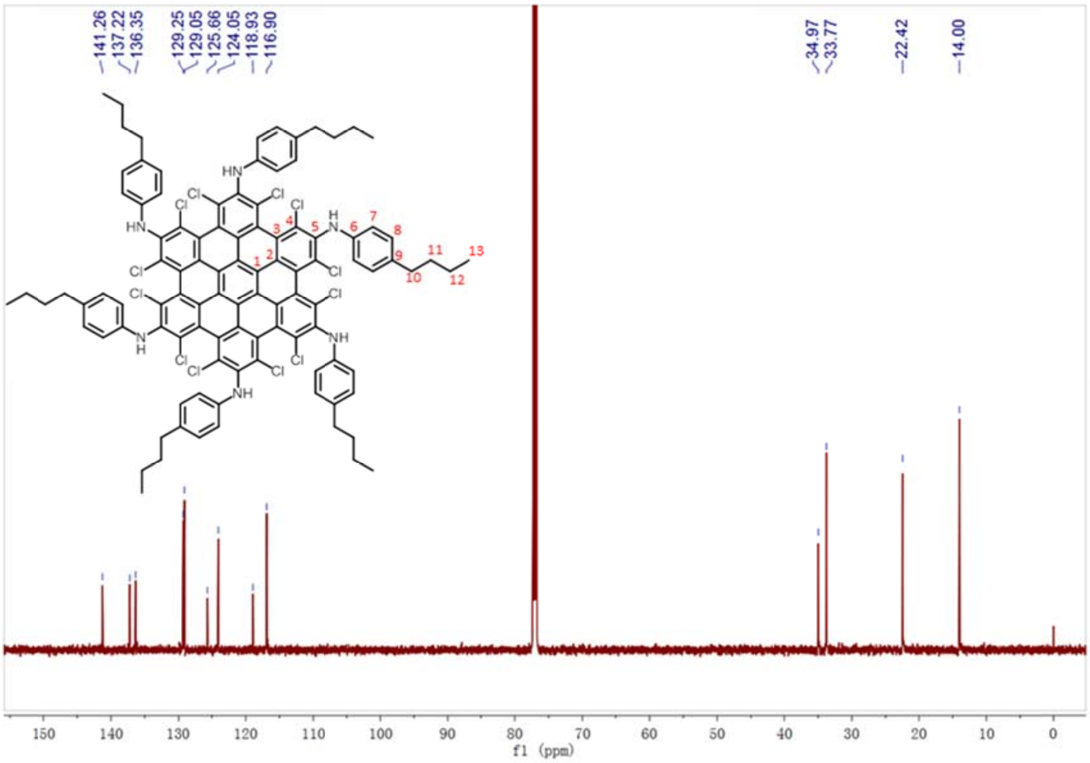

Supplementary Figure 14 ^{13}C NMR spectrum of **2c** in $CDCl_3$. ^{13}C NMR (151 MHz, $CDCl_3$) δ 141.26, 137.22, 136.35, 129.25, 129.05, 125.66, 124.05, 118.93, 116.90, 34.97, 33.77, 22.42, 14.00 ppm.

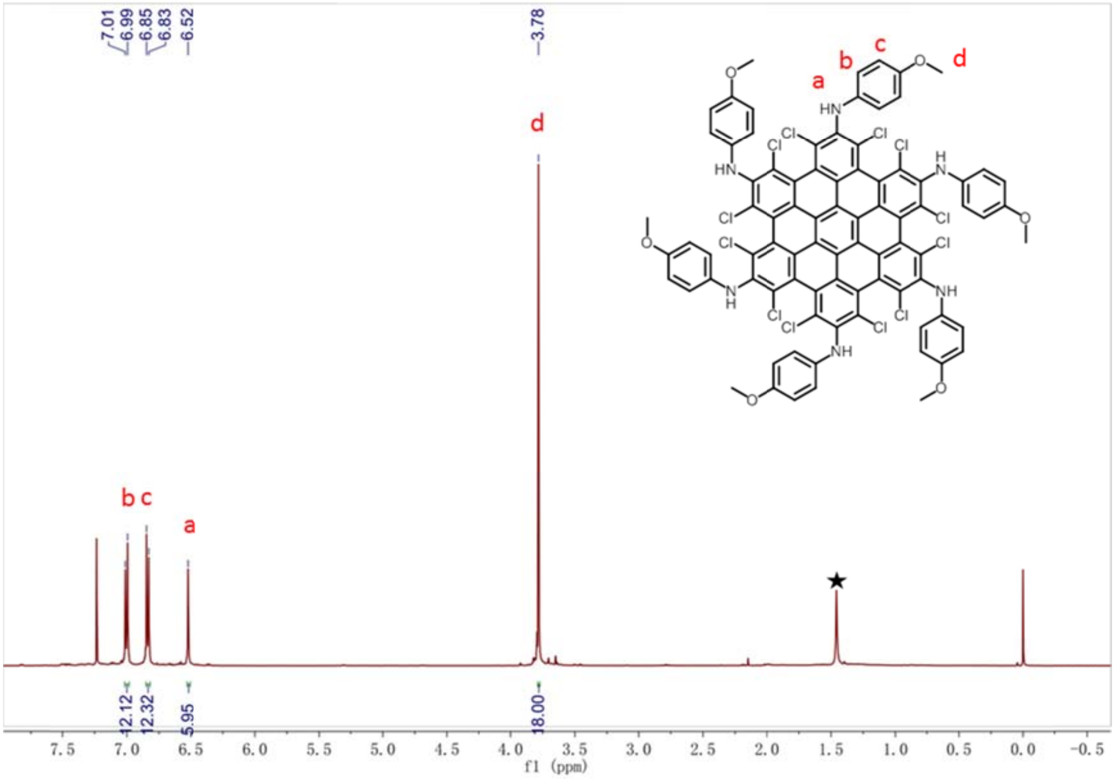

Supplementary Figure 15 ^{1}H NMR spectrum of **2d** in $CDCl_3$. The peaks at 1.47 ppm was assigned to the signal of water, marked by asterisk.^{1}H NMR (500 MHz, $CDCl_3$) δ 7.00 (d, J = 8.9 Hz, 12H), 6.84 (d, J = 8.9 Hz, 12H), 6.52 (s, 6H), 3.78 (s, 18H) ppm.

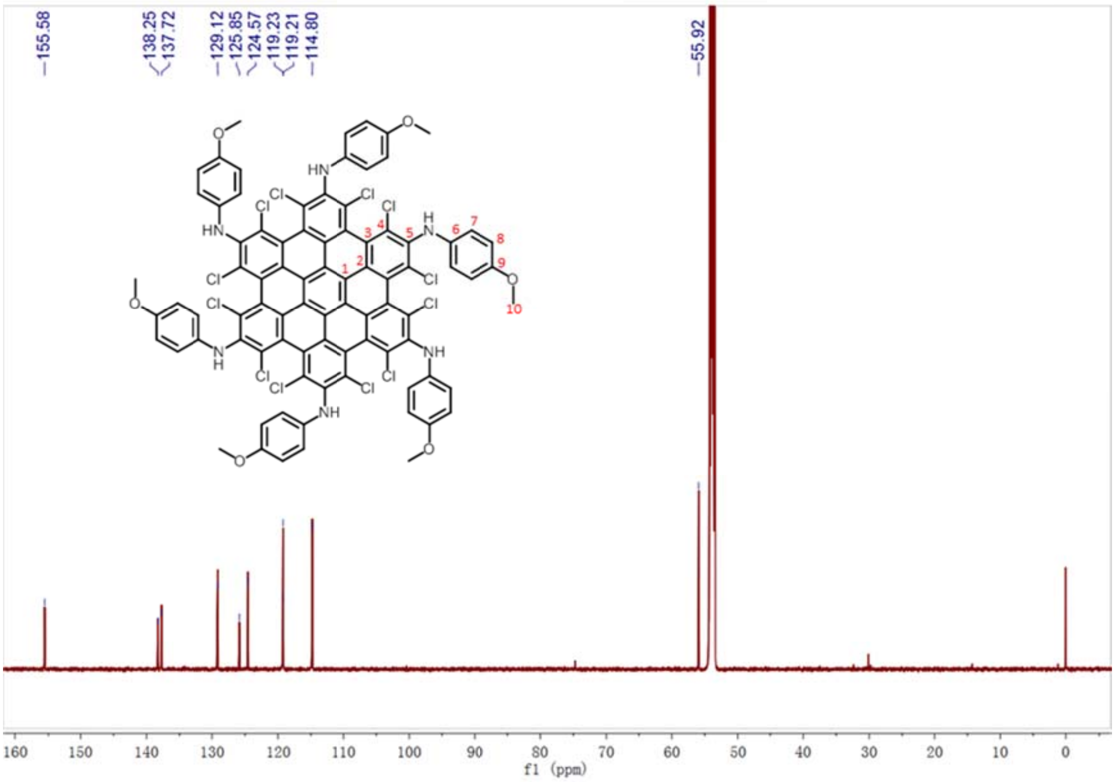

Supplementary Figure 16 ^{13}C NMR spectrum of **2d** in $CDCl_3$. ^{13}C NMR (151 MHz, CD_2Cl_2) δ 155.58, 138.25, 137.72, 129.12, 125.85, 124.57, 119.23, 119.21, 114.80, 55.92 ppm.

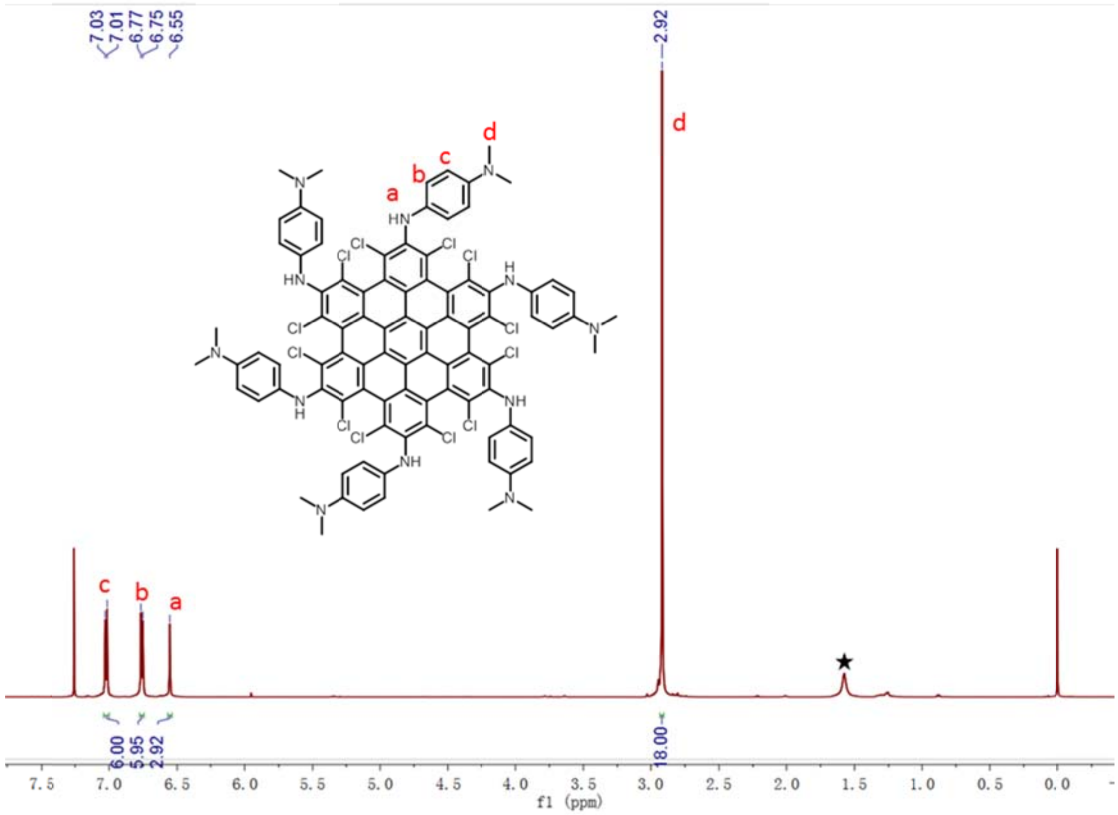

Supplementary Figure 17 ^{1}H NMR spectrum of **2e** in $CDCl_3$. ^{1}H NMR (600 MHz, $CDCl_3$) δ 7.02 (d, J = 8.8 Hz, 6H), 6.76 (d, J = 8.9 Hz, 6H), 6.55 (s, 3H), 2.92 (s, 18H) ppm.

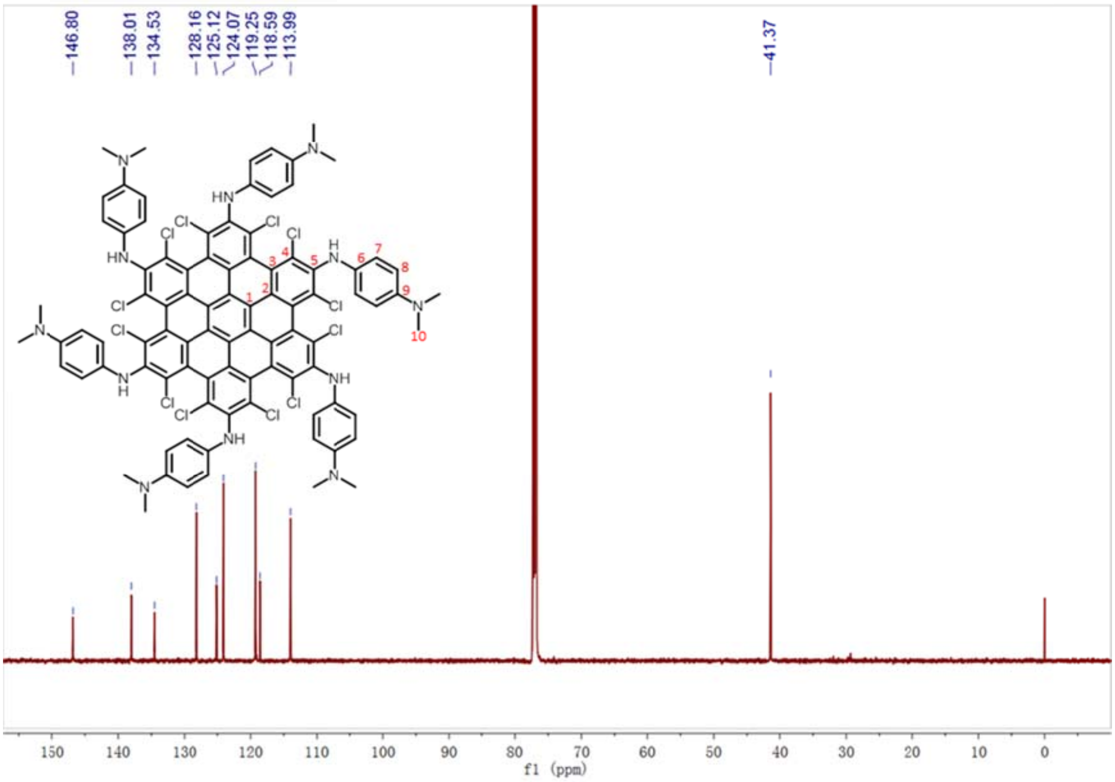

Supplementary Figure 18 ^{13}C NMR spectrum of **2e** in $CDCl_3$. ^{13}C NMR (151 MHz, $CDCl_3$) δ 146.80, 138.01, 134.53, 128.16, 125.12, 124.07, 119.25, 118.59, 113.99, 41.37 ppm.

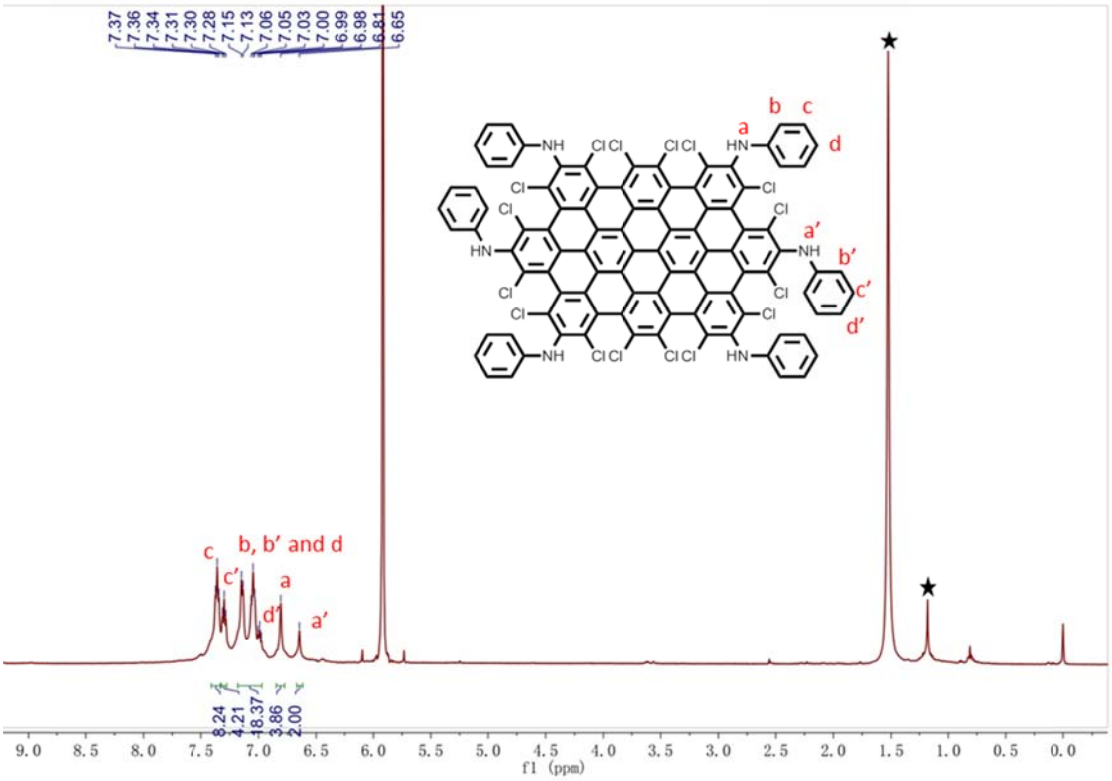

Supplementary Figure 19 ^{1}H NMR spectrum of **4a** in $C_2D_2Cl_4$. The peaks at 1.52 ppm and 1.23 ppm were assigned to the signals of water and hexanes, marked by asterisks. ^{1}H NMR (500 MHz, $C_2D_2Cl_4$): δ = 7.36 (t, J = 7.84 Hz, 8H), 7.30 (t, J = 7.88 Hz, 4H), 6.95-7.20 (m, 18H), 6.81 (s, 4H), 6.65 (s, 2H) ppm.

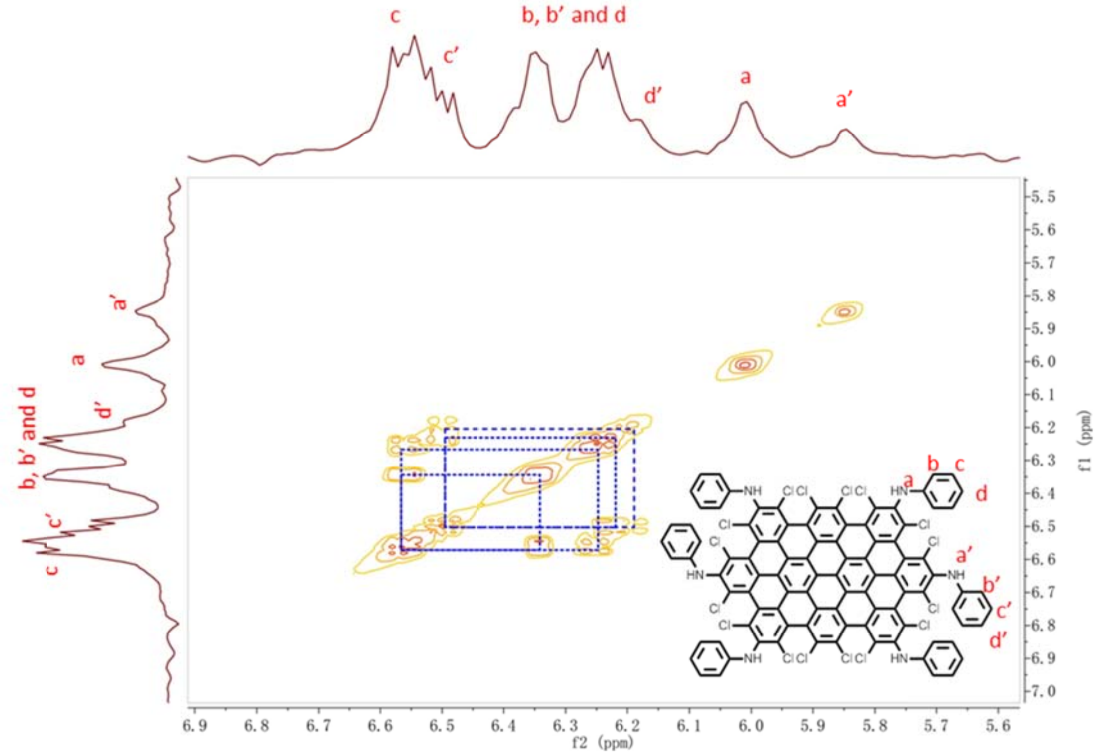

Supplementary Figure 20 ^{1}H-^{1}H COSY spectrum of **4a**

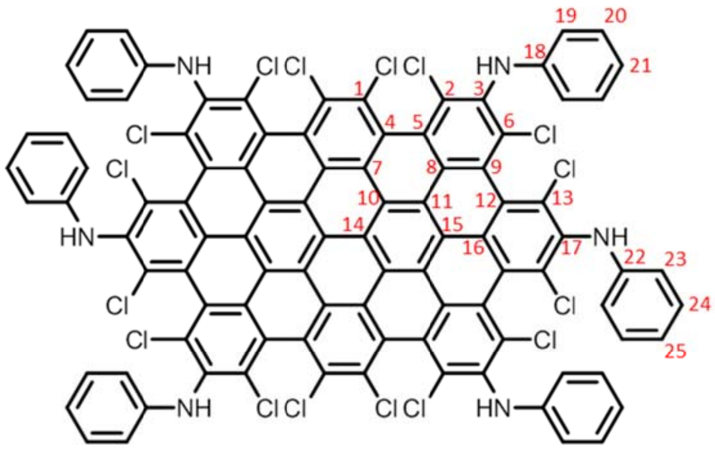

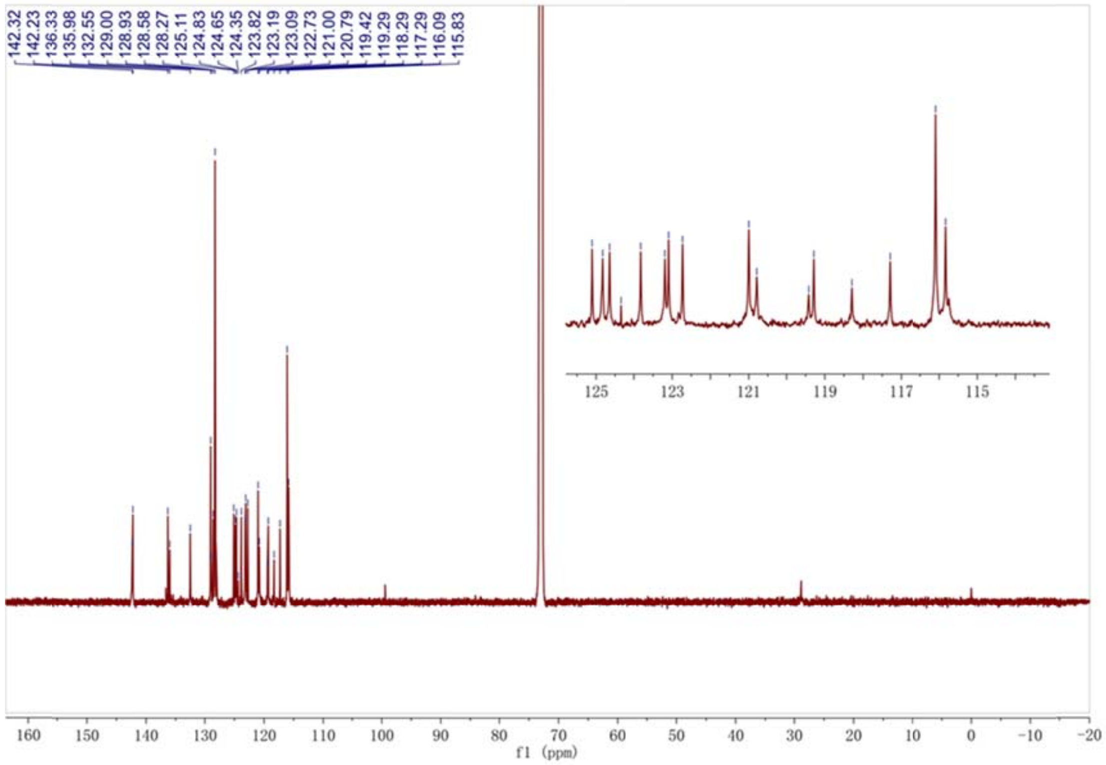

Supplementary Figure 21 ^{13}C NMR spectrum of **4a** in $C_2D_2Cl_4$. ^{13}C NMR (151 MHz, $C_2D_2Cl_4$) δ 142.32, 142.23, 136.33, 135.98, 132.55, 129.00, 128.93, 128.58, 128.27, 125.11, 124.83, 124.65, 124.35, 123.82, 123.19, 123.09, 122.73, 121.00, 120.79, 119.42, 119.29, 118.29, 117.29, 116.09, 115.83 ppm.

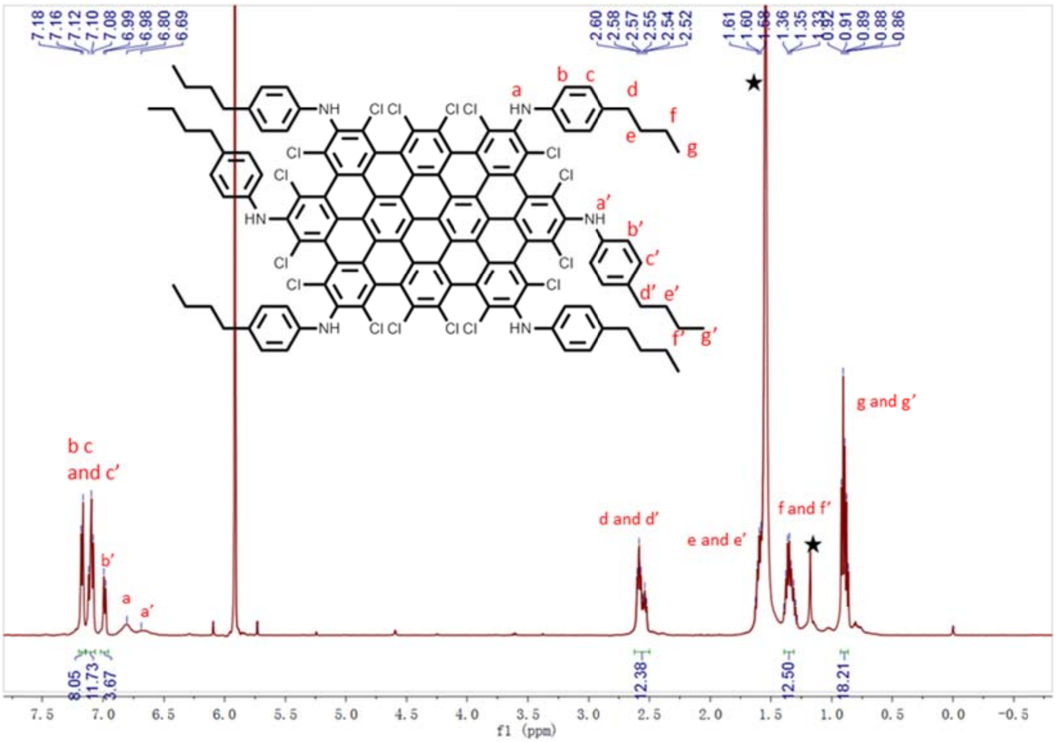

Supplementary Figure 22 ^{1}H NMR spectrum of **4b** in $C_2D_2Cl_4$. The peaks at 1.55 ppm and 1.22 ppm were assigned to the signals of water and hexanes, marked by asterisks. ^1H NMR (500 MHz, $C_2D_2Cl_4$) δ 7.17 (d, J = 8.0 Hz, 8H), 7.14 – 7.07 (m, 12H), 6.99 (d, J = 8.0 Hz, 4H), 2.62 – 2.50 (m, 12H), 1.39 – 1.31 (m, 13H), 0.93 – 0.87 (m, 18H). ppm.

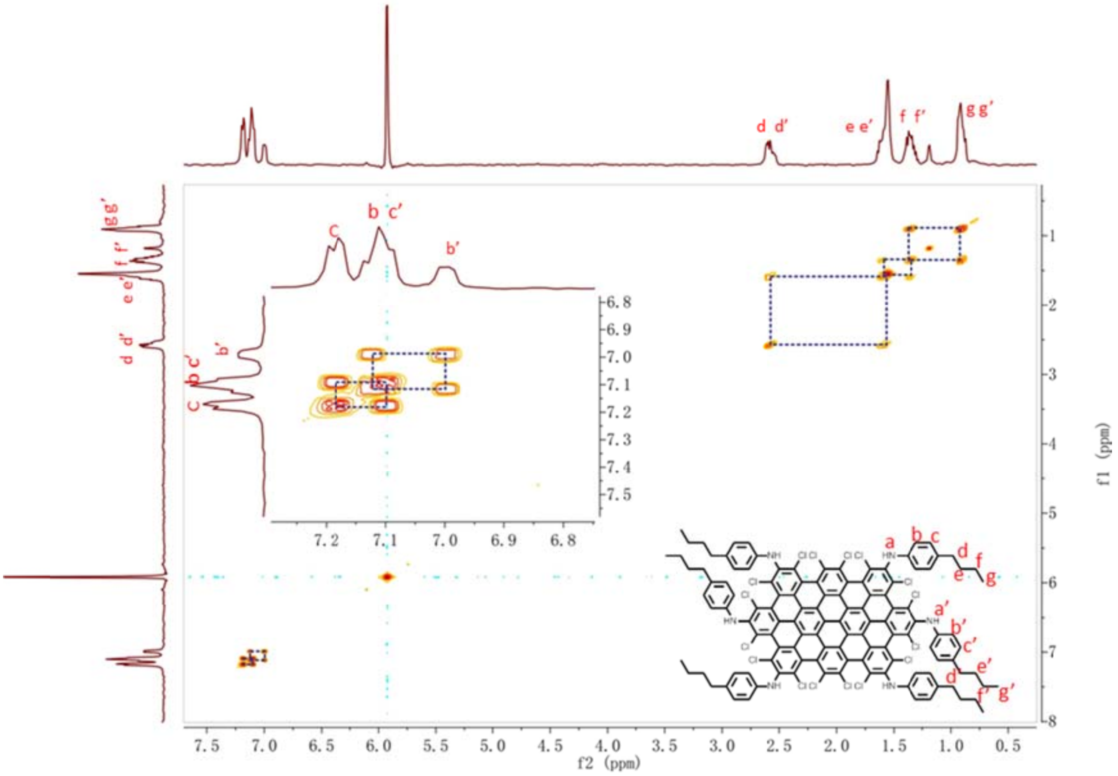

Supplementary Figure 23 ^1H-^1H COSY spectrum of **4b** in $C_2D_2Cl_4$

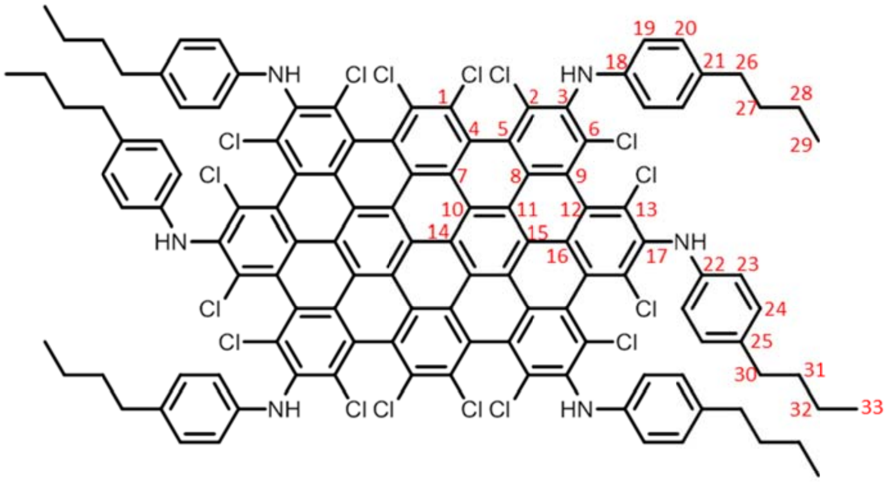

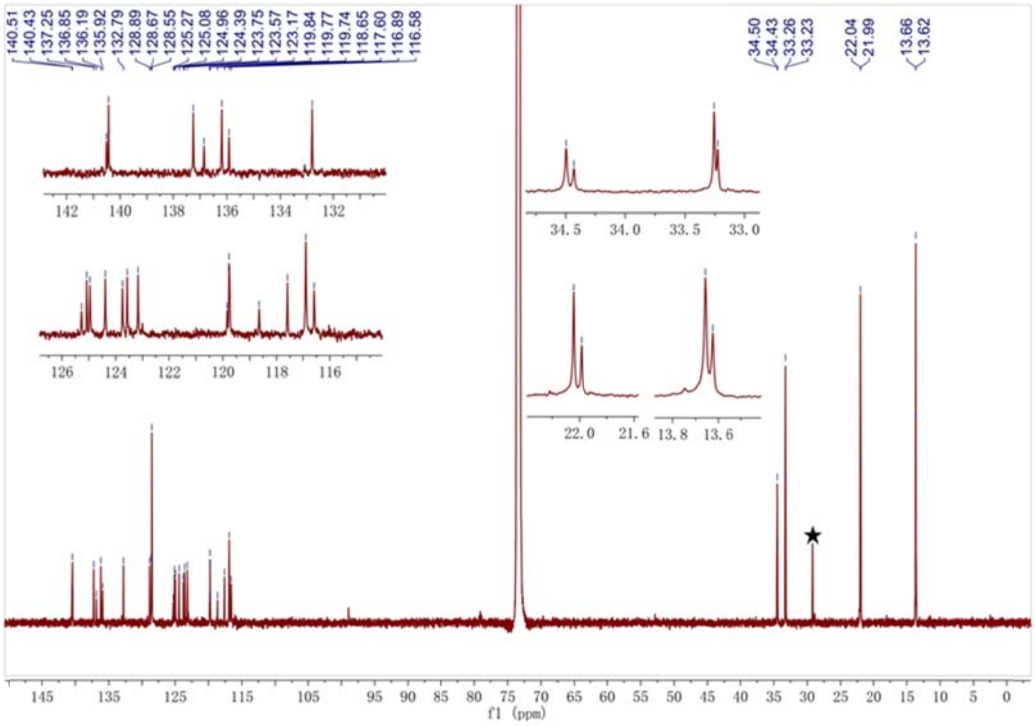

Supplementary Figure 24 ^{13}C NMR spectrum of **4b** in $C_2D_2Cl_4$. The peaks at 29.54 ppm was assigned to the signal of hexane, marked by asterisk. ^{13}C NMR (151 MHz, $C_2D_2Cl_4$) δ 140.51, 140.43, 137.25, 136.85, 136.19, 135.92, 132.79, 128.89, 128.67, 128.55, 125.27, 125.08, 124.96, 124.39, 123.75, 123.57, 123.17, 119.84, 119.77, 119.74, 118.65, 117.60, 116.89, 116.58, 34.50, 34.43, 33.26, 33.23, 22.04, 21.99, 13.66, 13.62 ppm.

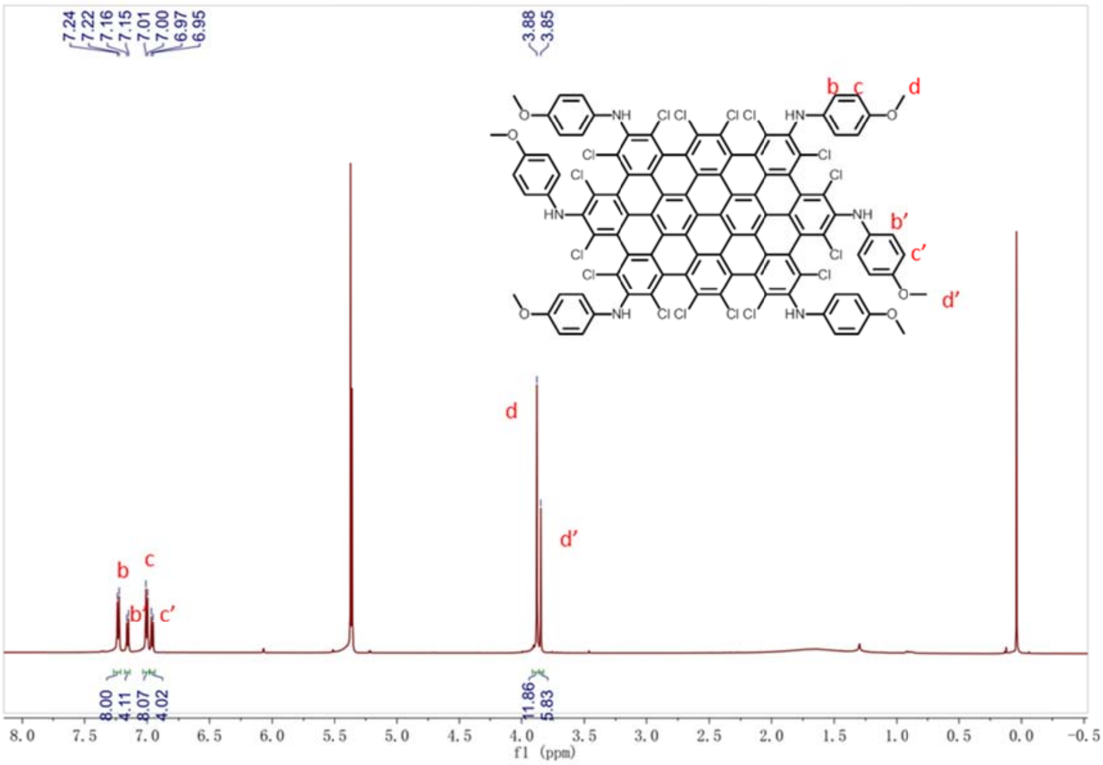

Supplementary Figure 25 ^{1}H NMR spectrum of **4c** in CD_2Cl_2. ^{1}H NMR (600 MHz, CD_2Cl_2) δ 7.19 (d, J = 8.8 Hz, 8H), 7.12 (d, J = 8.8 Hz, 4H), 6.96 (d, J = 8.8 Hz, 8H), 6.92 (d, J = 8.9 Hz, 4H), 3.84 (s, 12H), 3.81 (s, 6H) ppm.

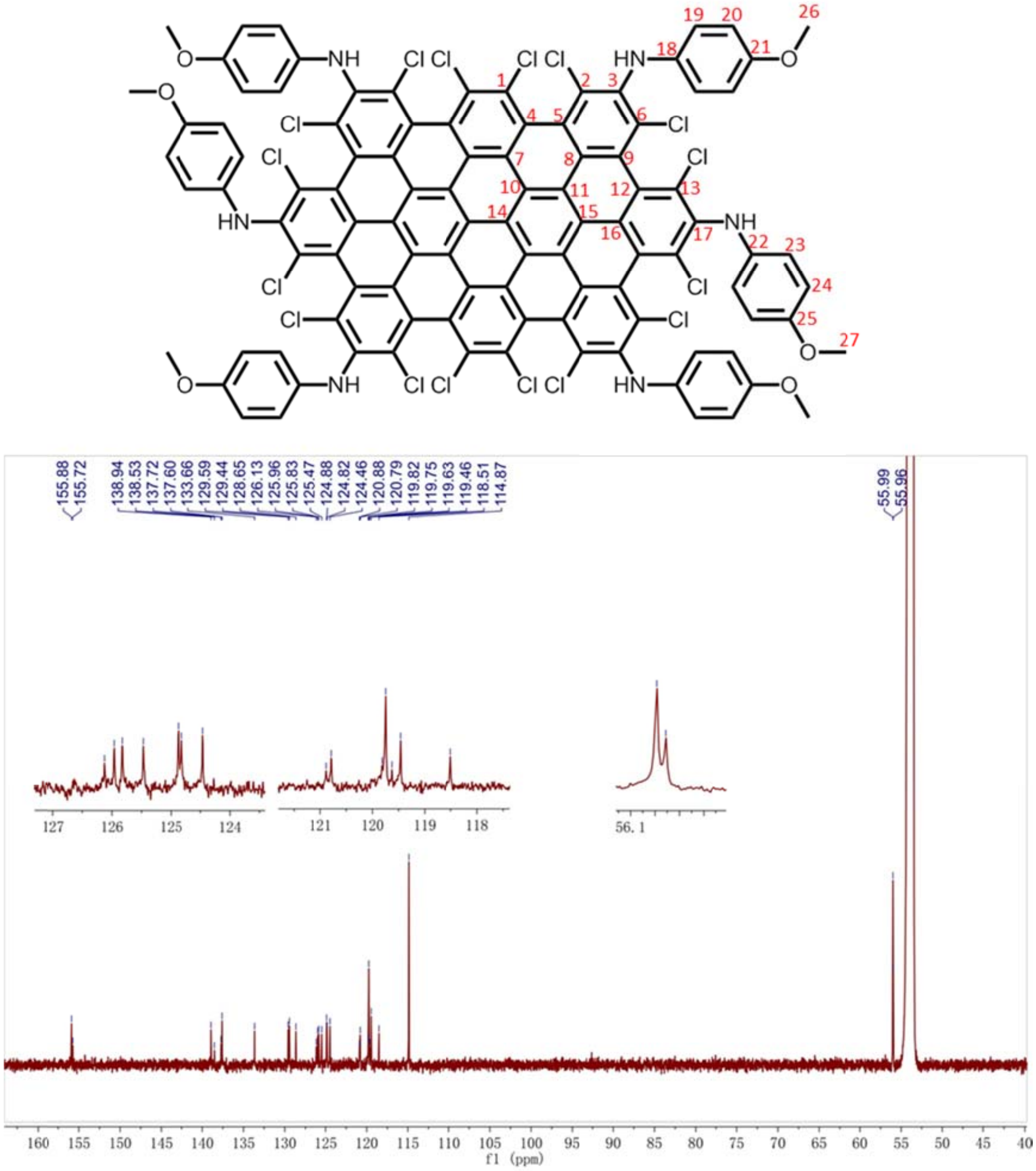

Supplementary Figure 26 ^{13}C NMR spectrum of **4c** in CD_2Cl_2. ^{13}C NMR (151 MHz, CD_2Cl_2) δ 155.88, 155.72, 138.94, 138.53, 137.72, 137.60, 133.66, 129.59, 129.44, 128.65, 126.13, 125.96, 125.83, 125.47, 124.88, 124.82, 124.46, **1**20.88, 120.79, 119.82, 119.75, 119.63, 119.46, 118.51, 114.87, 55.99, 55.96 ppm.

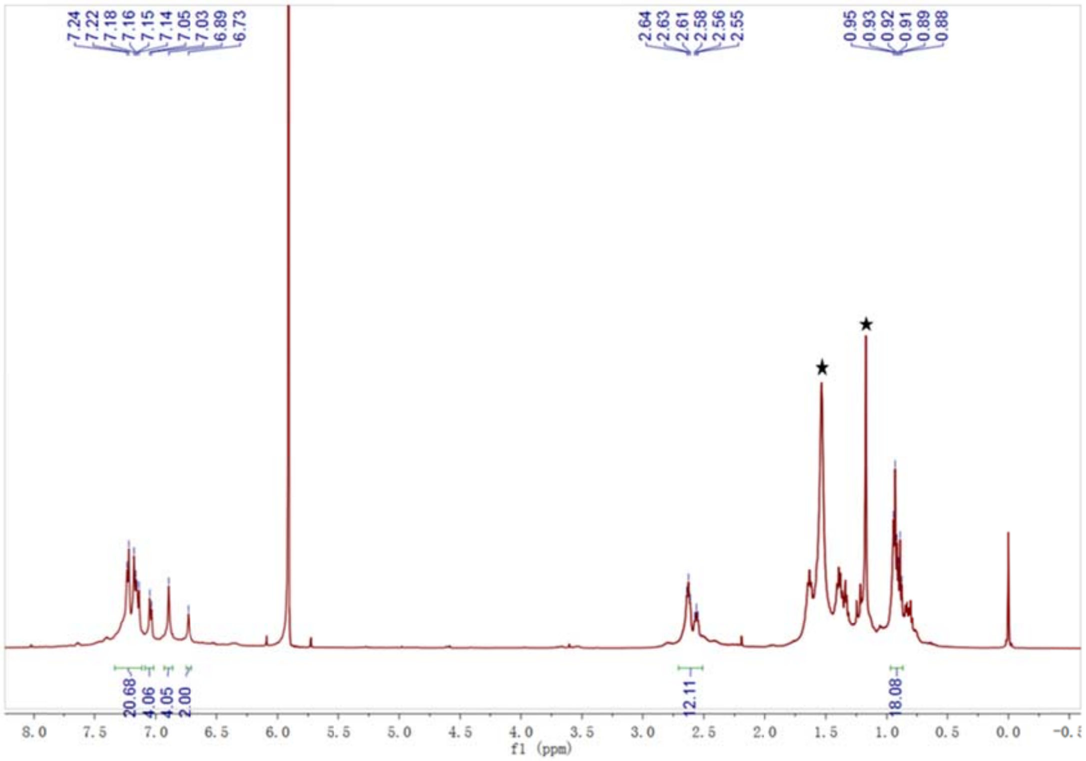

Supplementary Figure 27 ^{1}H NMR spectrum of **6a** in $C_2D_2Cl_4$. The peaks at 1.53 ppm and 1.24 ppm were assigned to the signals of water and hexanes in the solvent, marked by asterisks. ^{1}H NMR (500 MHz, $C_2D_2Cl_4$): δ = 7.11-7.27 (m, 20H), 7.04 (d, J = 7.19 Hz, 4H,), 6.89 (s, 4H), 6.73 (s, 2H), 2.55-2.64 (m, 12H), 0.88-0.95 (m, 18H) ppm. Based on the ^{1}H NMR spectrum of **6a**, two kinds of anilino groups with a ratio of 1:2 can be distigiuished, suggesting the C_{2v} symmetry of **6a**.

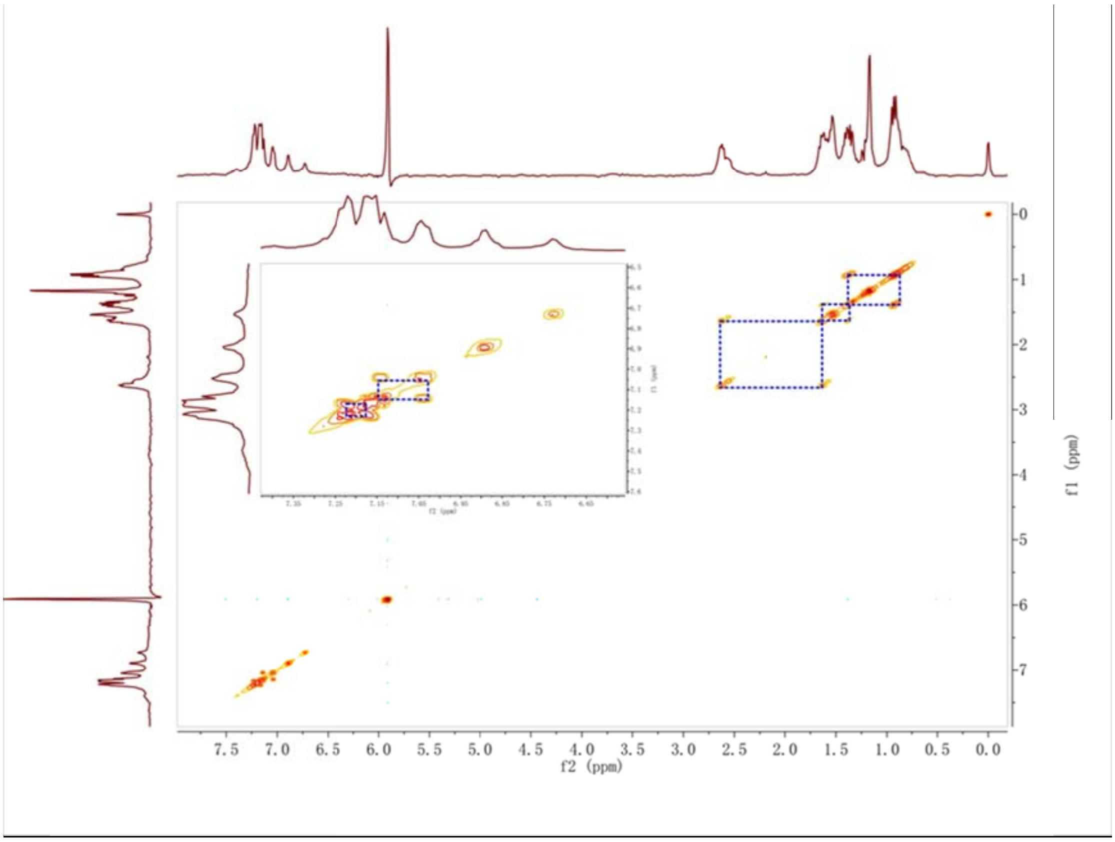

Supplementary Figure 28 ^{1}H-^{1}H COSY spectrum of **6a** in $C_2D_2Cl_4$. ^{1}H-^{1}H COSY spectrum **6a** confirmed two kinds of anilino groups with a ratio of 1:2 **in 6a**.

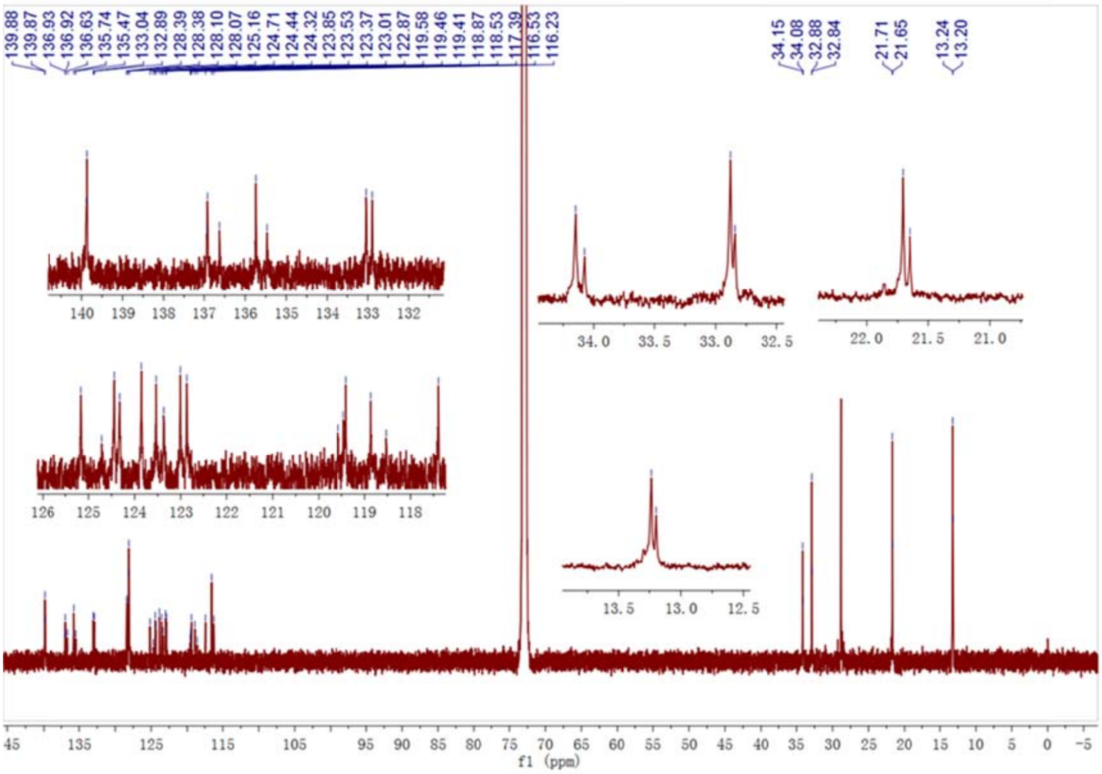

Supplementary Figure 29 ^{13}C NMR spectrum of **6a** in $C_2D_2Cl_4$. The peaks at 28.87 ppm was assigned to the signal of hexanes, marked by asterisk. ^{13}C NMR (151 MHz, $C_2D_2Cl_4$) δ 139.88, 139.87, 136.93, 136.92, 136.63, 135.74, 135.47, 133.04, 132.89, 128.39, 128.38, 128.10, 128.07, 125.16, 124.71, 124.44, 124.32, 123.85, 123.53, 123.37, 123.01, 122.87, 119.58, 119.46, 119.41, 118.87, 118.53, 117.39, 116.53, 116.23, 34.15, 34.08, 32.88, 32.84, 21.71, 21.65, 13.24, 13.20 ppm. ^{13}C NMR spectrum of **6a** validated the C_{2v} symmetry of **6a**.

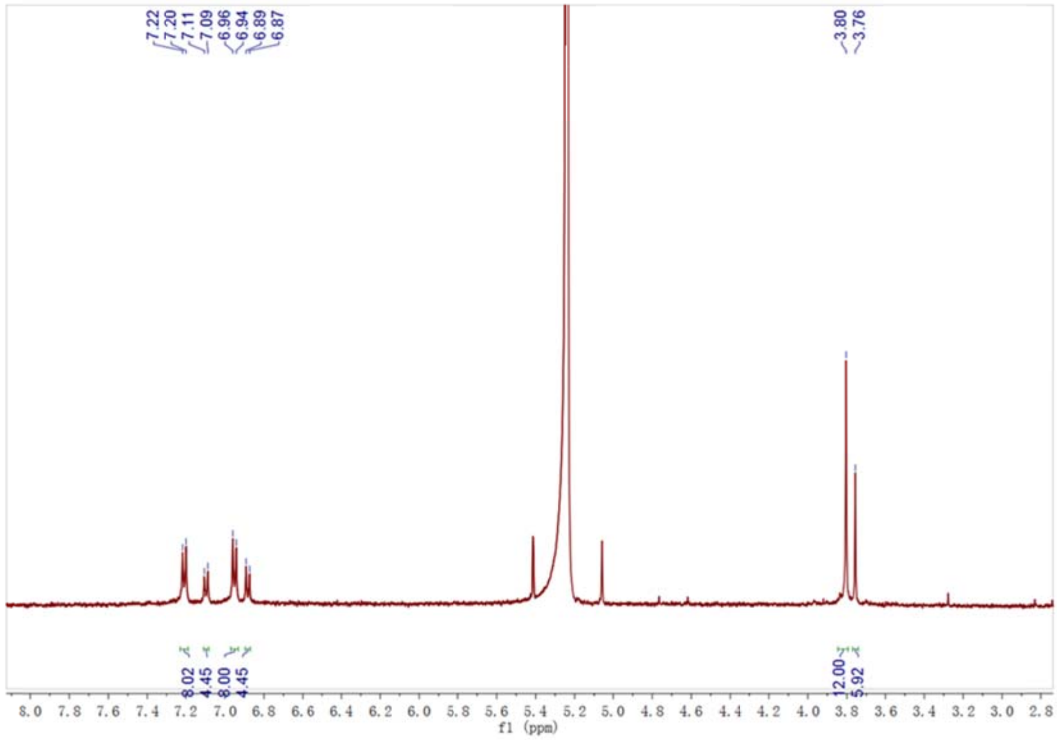

Supplementary Figure 30 ^{1}H NMR spectrum of **6b** in CD_2Cl_2 ^{1}H-NMR (500 MHz, CD_2Cl_2): δ = 7.21 (8H, d, J = 9.95 Hz), 7.10 (d, J = 9.17 Hz, 4H), 6.95 (d, J = 8.00 Hz, 8H), 6.88 (d, J = 8.19 Hz, 4H), 6.88(t, J = 7.46 Hz, 6H), 3.80(s, 12H), 3.76(s, 6H) ppm. Based on the ^{1}H NMR spectrum of **6b**, two kinds of anilino groups with a ratio of 1:2 can be distinguished, suggesting the C_{2v} symmetry of **6b**.

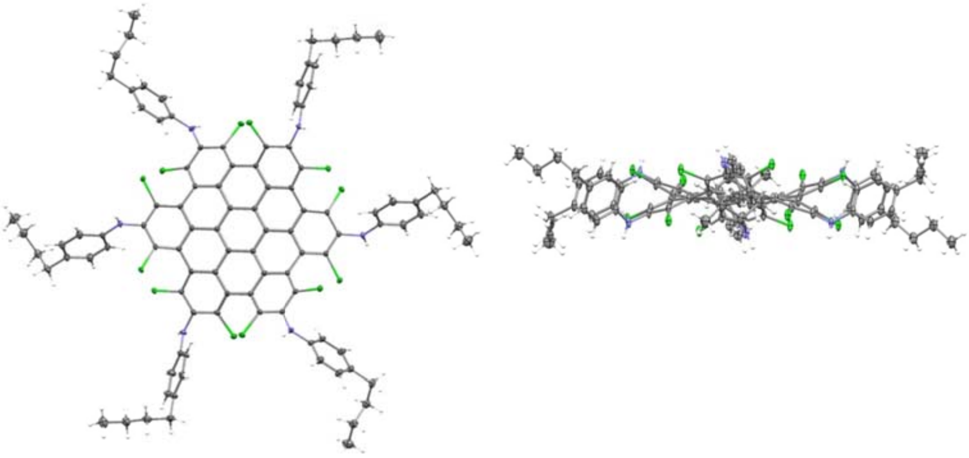

Supplementary Figure 31 The crystal structure of **2c**

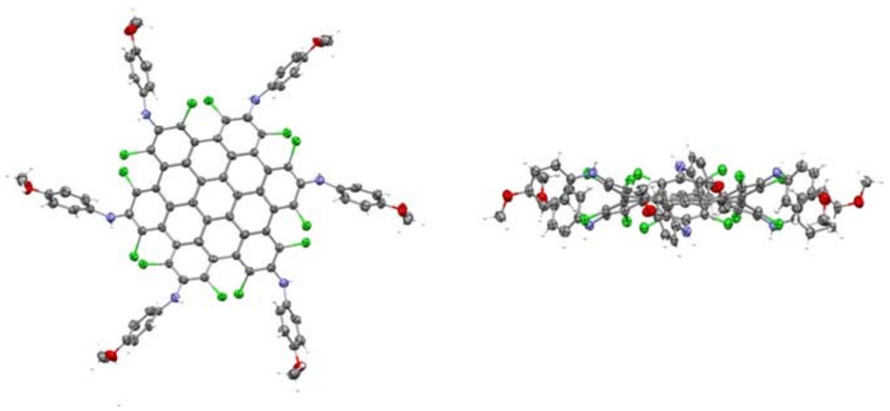

Supplementary Figure 32 The crystal structure of **2d**

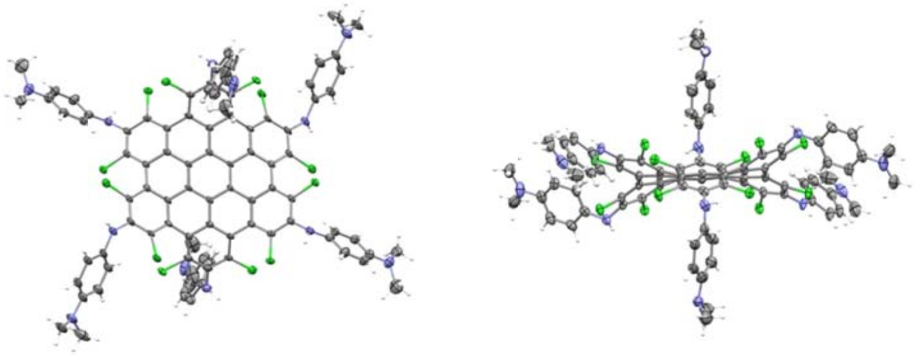

Supplementary Figure 33 The crystal structure of **2e**

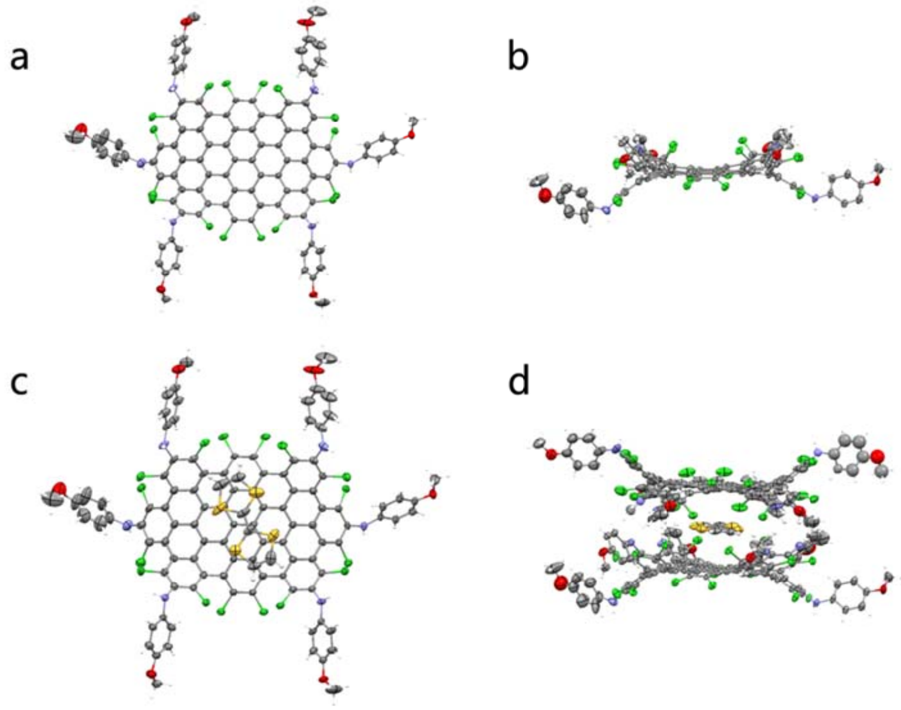

Supplementary Figure 34 The crystal structure of **4c**⊃TTF.

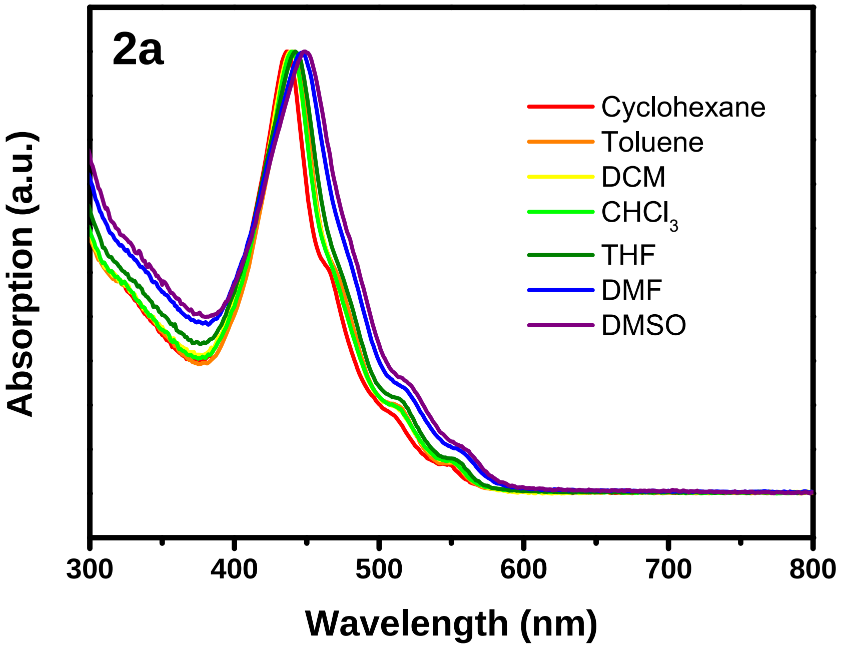

Supplementary Figure 35 UV-Vis spectra of **2a** in various solvents with increasing polarity (the concentration of the solution is 5×10^{-6} mol L^{-1}). The redshift is about 13 nm from the most nonpolar solvent (cyclohexane) to the most polar solvent (DMSO).

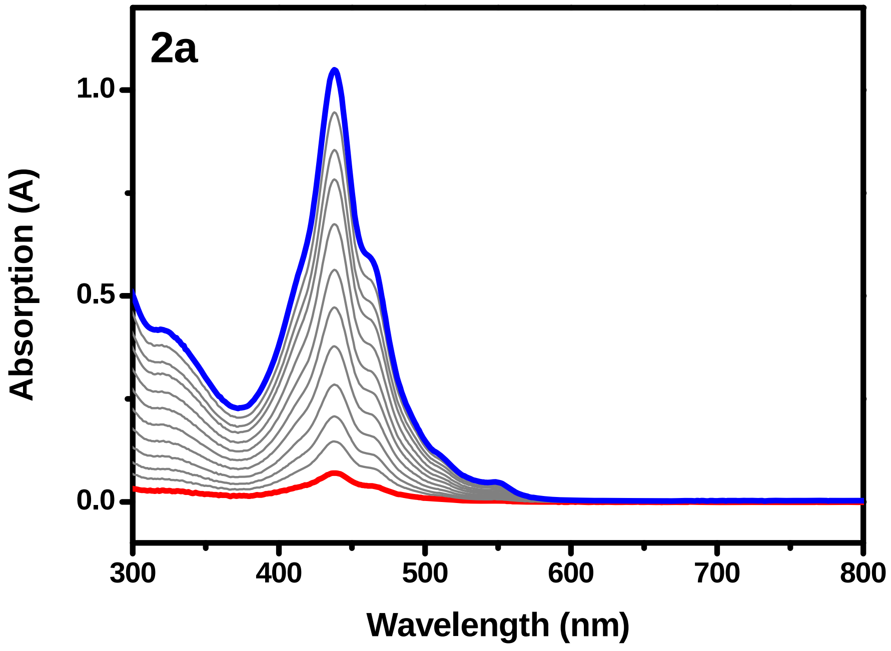

Supplementary Figure 36 UV-Vis spectra of **2a** at variable concentrations from 1.1×10^{-6} mol L^{-1} to 16.7×10^{-6} mol L^{-1} in DCM solution.

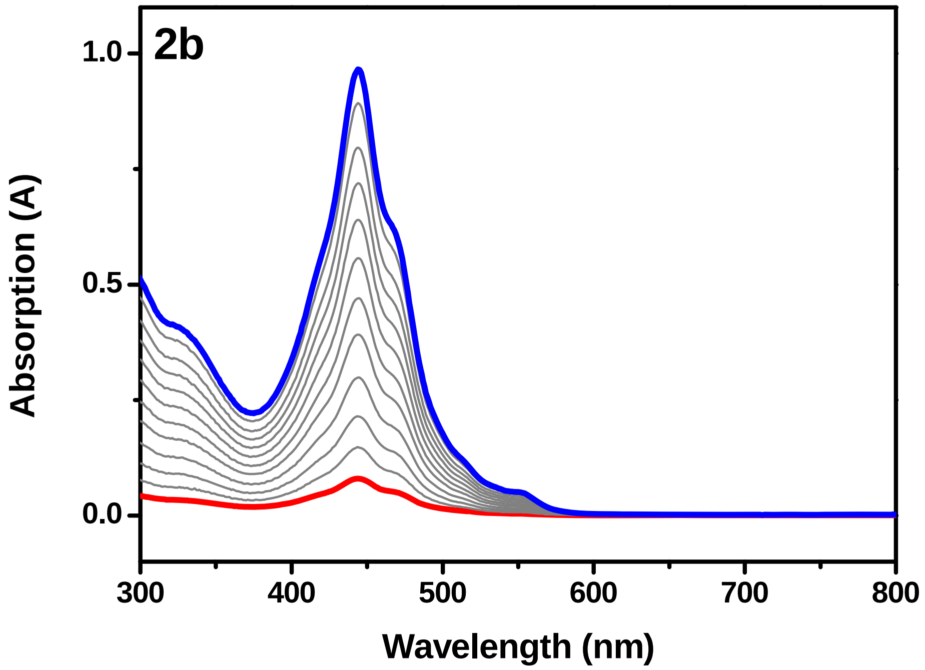

Supplementary Figure 37 UV-Vis spectra of **2b** at variable concentrations from 1.1×10^{-6} mol L^{-1} to 13.2×10^{-6} mol L^{-1} in DCM solution.

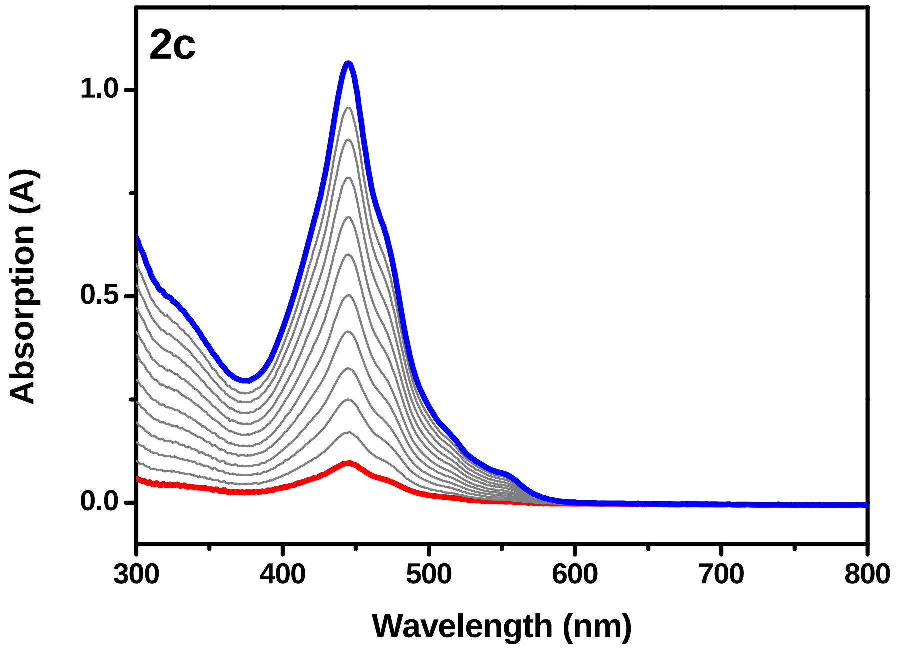

Supplementary Figure 38 UV-Vis spectra of **2c** at variable concentrations from 1.0×10^{-6} mol L^{-1} to 11.7×10^{-6} mol L^{-1} in DCM solution.

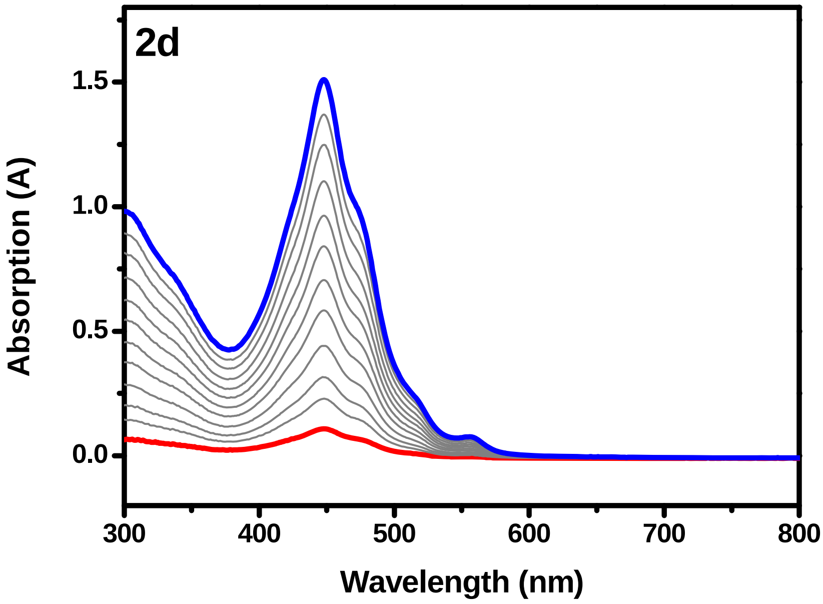

Supplementary Figure 39 UV-Vis spectra of **2d** at variable concentrations from 1.2×10^{-6} mol L^{-1} to 14.2×10^{-6} mol L^{-1} in DCM solution.

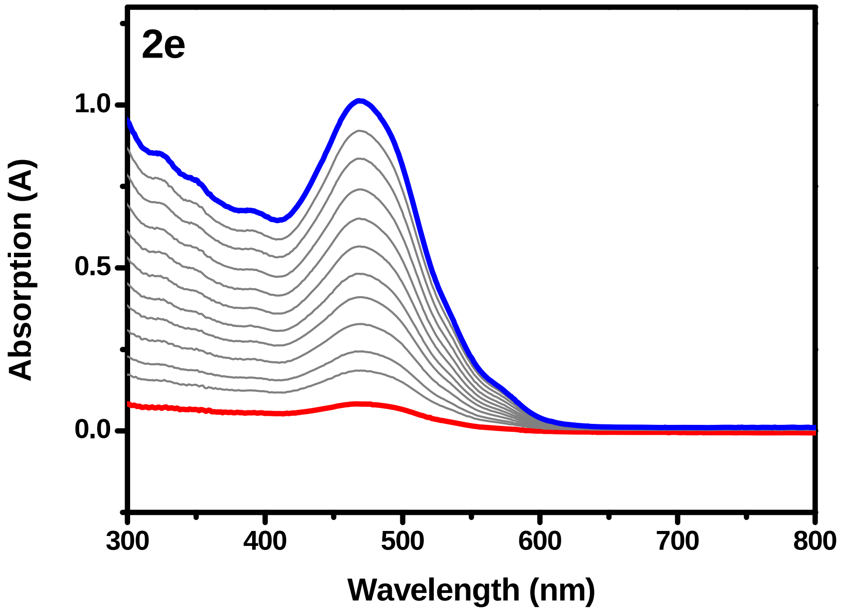

Supplementary Figure 40 UV-Vis spectra of **2e** at variable concentrations from 0.6×10^{-6}mol L^{-1} to 7.2×10^{-6} mol L^{-1} in DCM solution.

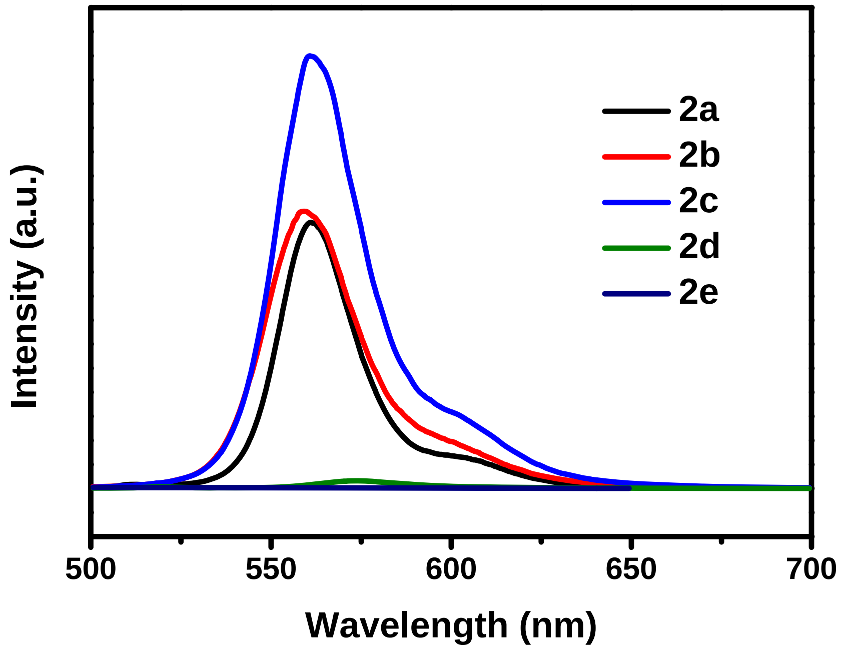

Supplementary Figure 41 Photoluminescence spectra of **2a** (black), **2b** (red), **2c** (blue), **2d** (olive), and **2e** (navy) in DCM (concentration is 2×10^{-6}mol L^{-1}).

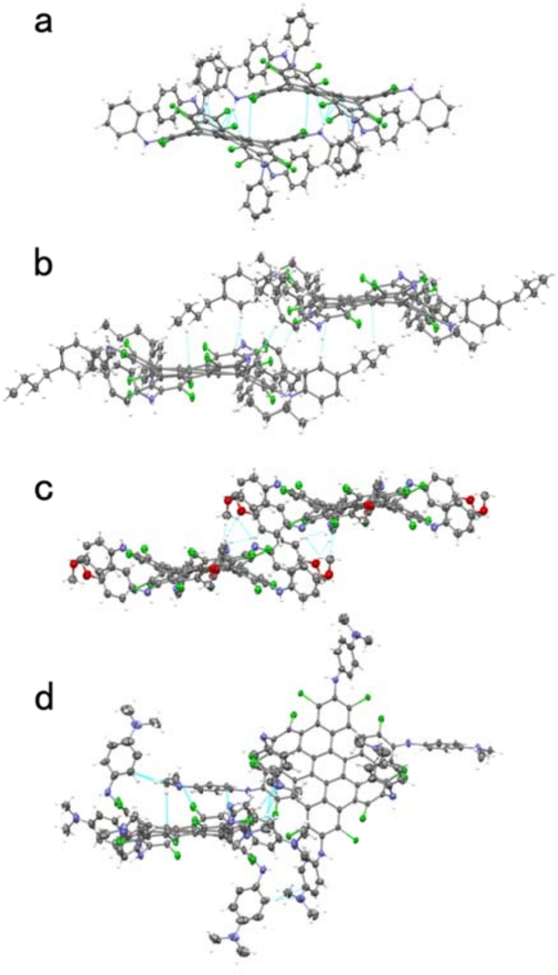

Supplementary Figure 42 Intermolecular interactions (represented by dashed cyan lines) in the crystal packing of **2a** (a), **2c** (b), **2d** (c) and **2e** (d).

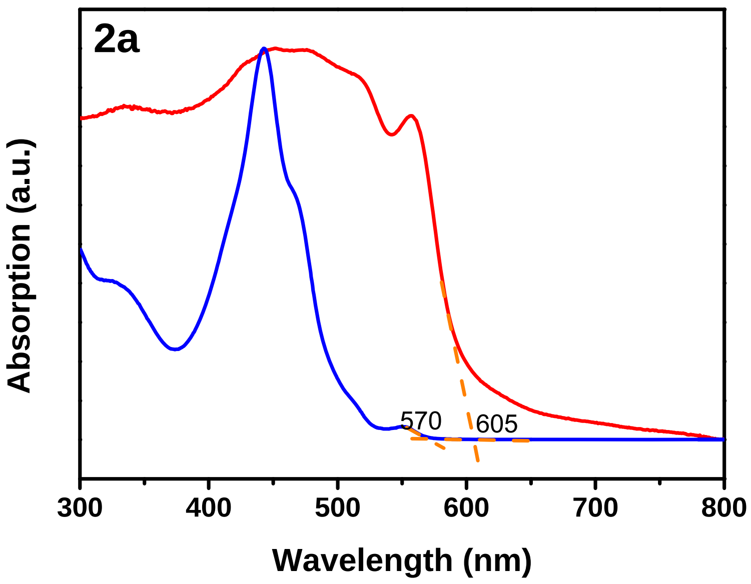

Supplementary Figure 43 UV-Vis spectra of **2a** in the solid state (red) and in DCM solution (blue, 10^{-5} mol·L^{-1}).

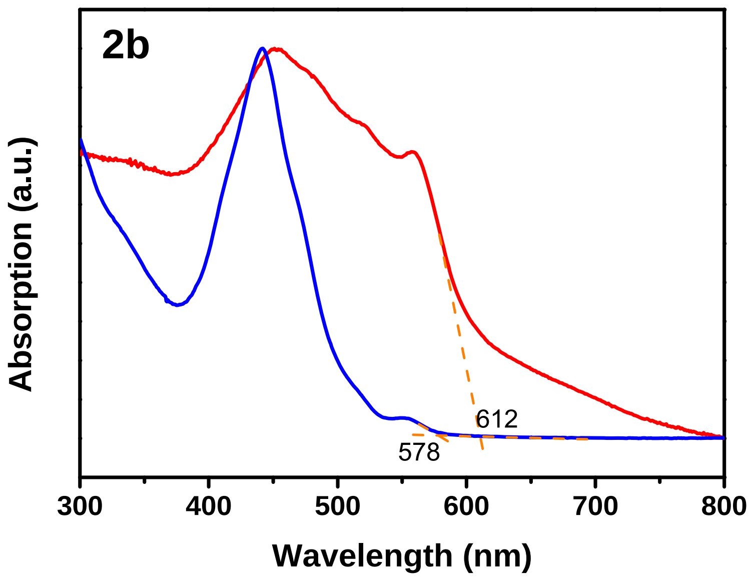

Supplementary Figure 44 UV-Vis spectra of **2b** in the solid state (red) and in DCM solution (blue, 10^{-5} mol·L^{-1}).

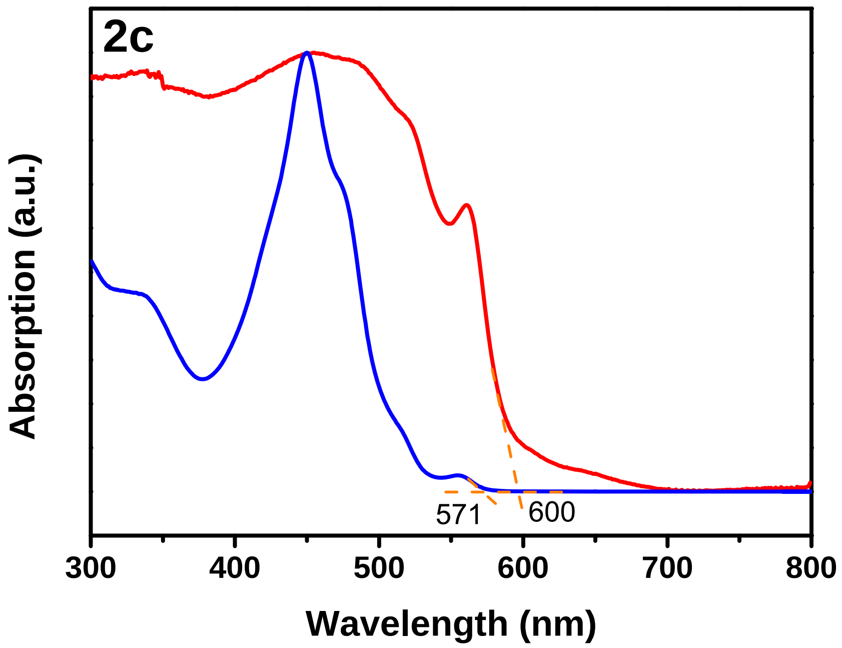

Supplementary Figure 45 UV-Vis spectra of **2c** in the solid state (red) and in DCM solution (blue, 10^{-5} mol·L^{-1}).

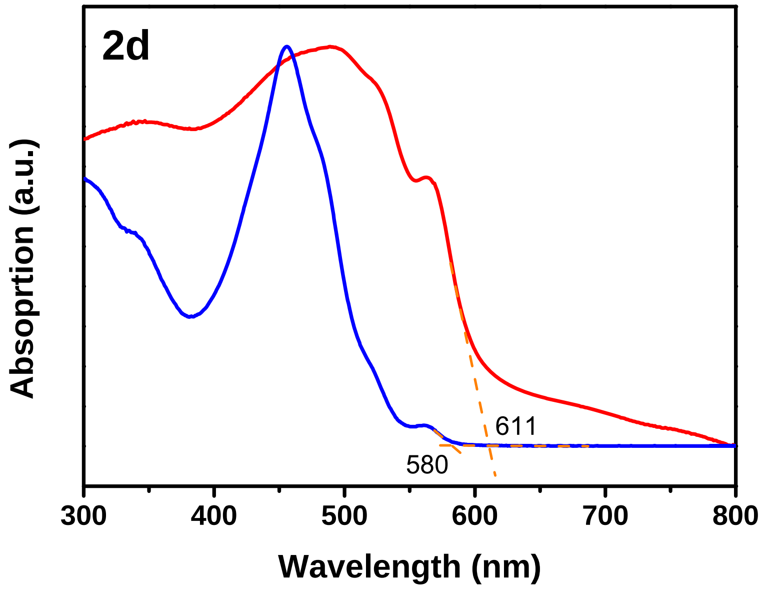

Supplementary Figure 46 UV-Vis spectra of **2d** in the solid state (red) and in DCM solution (blue, 10^{-5} mol·L^{-1}).

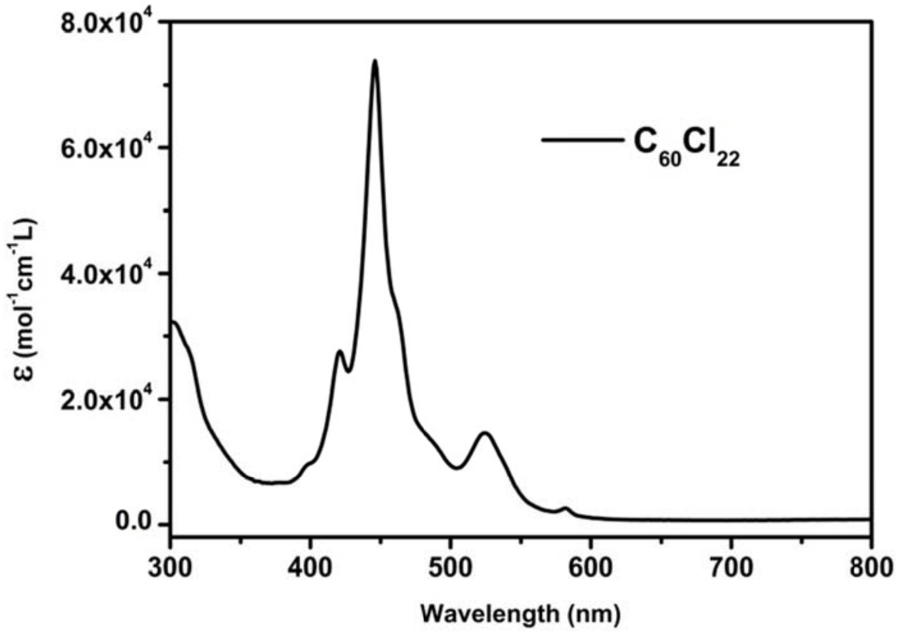

Supplementary Figure 47 UV-Vis absorption spectra of **3** ($C_{60}Cl_{22}$) in DCM solution (10^{-5} mol L^{-1}).

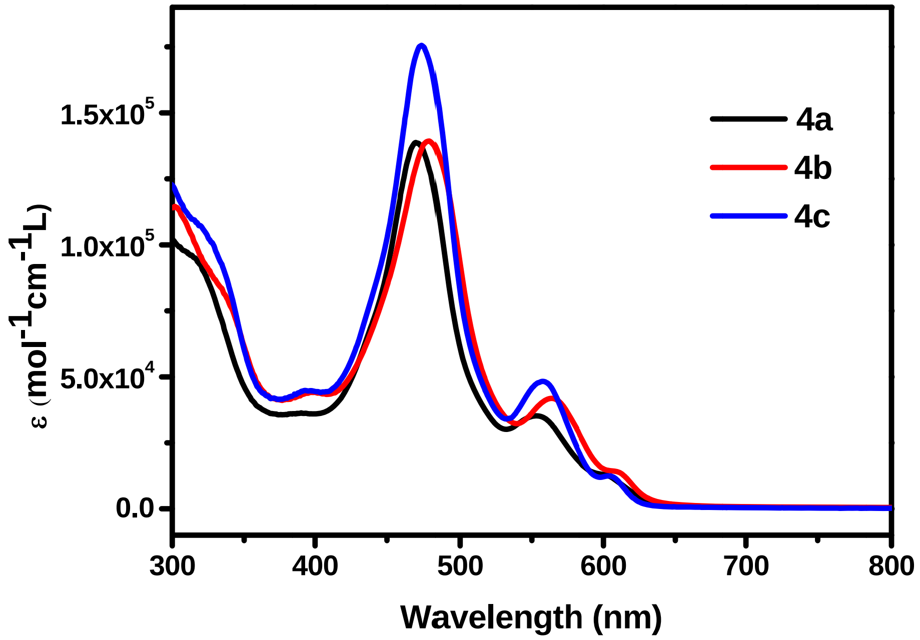

Supplementary Figure 48 UV-Vis absorption spectra of **4a** (black), **4b** (red) and **4c** (blue) in DCM solution (10^{-5} mol L^{-1}).

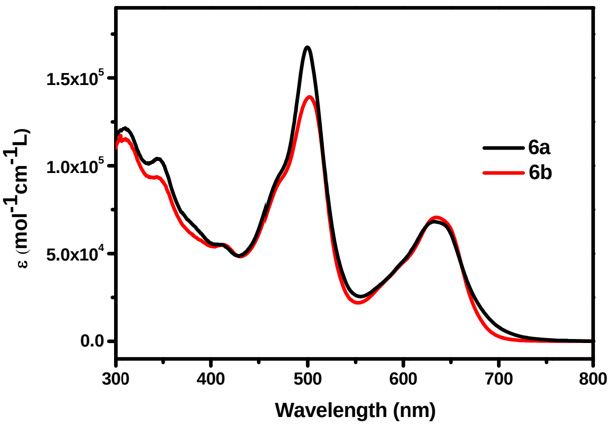

Supplementary Figure 49 UV-Vis absorption spectra of **6a** (black) and **6b** (red) in DCM solution (10^{-5} mol L^{-1}).

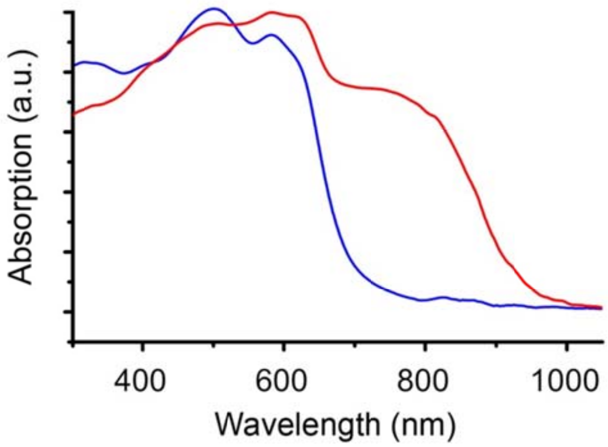

Supplementary Figure 50 UV-Vis-NIR spectra of **4c** and 2·**4c**⊃TTF in the solid state acquired in a diffuse-reflectance mode.

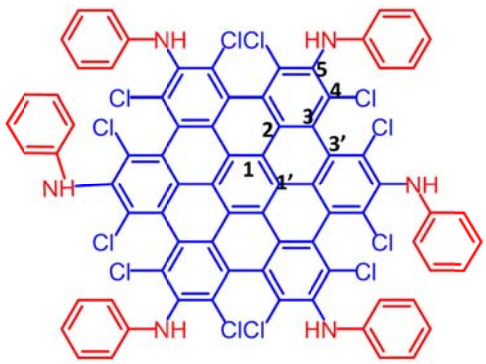

Supplementary Figure 51 Numbering of the carbon atoms of **2a**

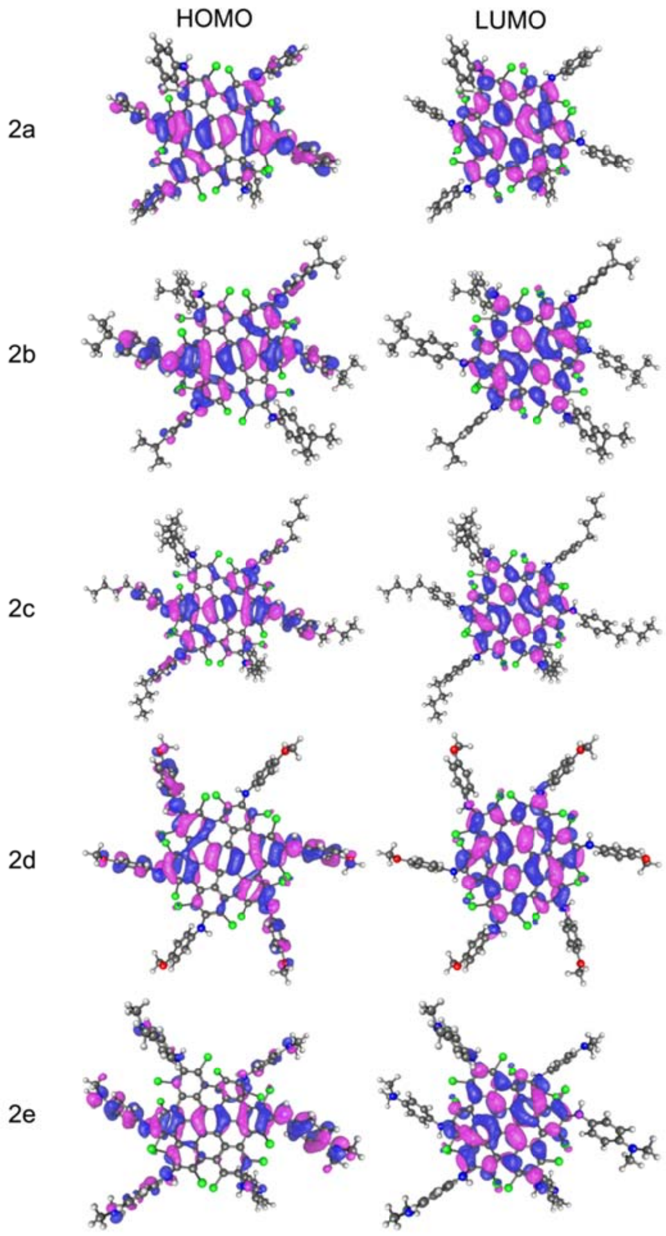

Supplementary Figure 52 Visualization of frontier molecular orbitals of **2**

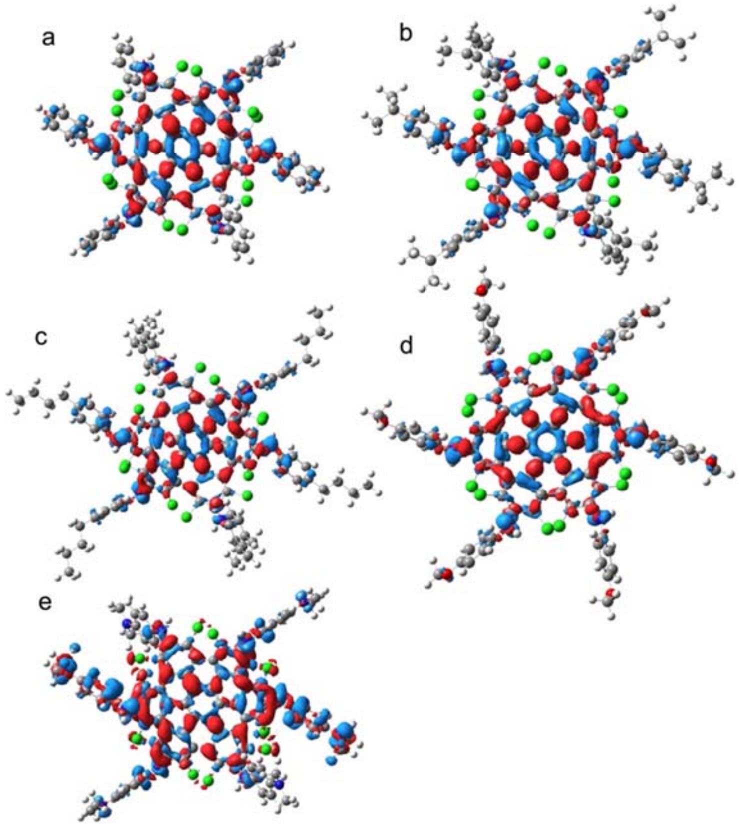

Supplementary Figure 53 The electron density differences between the first excitation state and the ground state of **2** (a, **2a**, b, **2b**, c, **2c**, d, **2d** and e, **2e**). (Blue and red refer to a decrease and an increase in electron density, respectively)

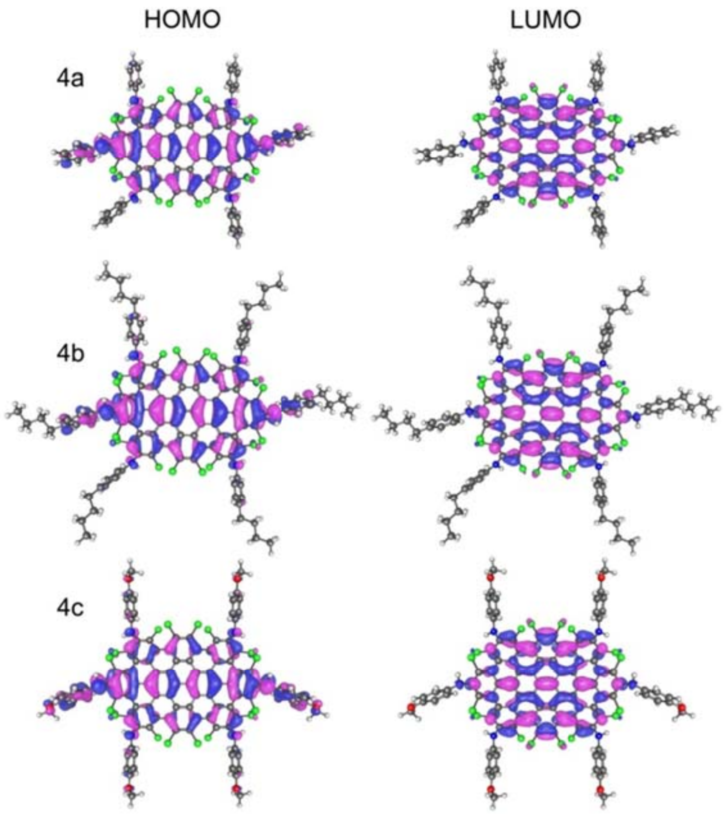

Supplementary Figure 54 Visualization of frontier molecular orbitals of **4**

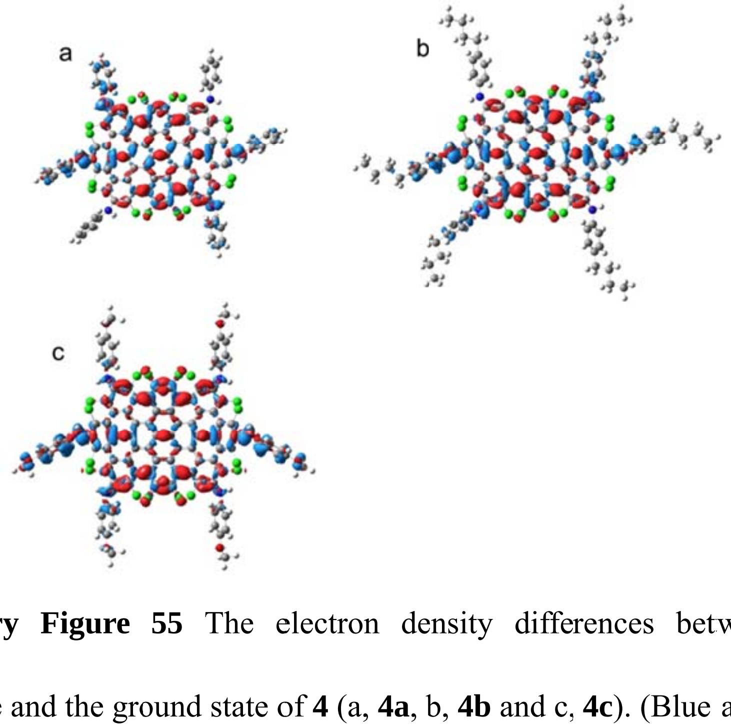

Supplementary Figure 55 The electron density differences between the first excitation state and the ground state of **4** (a, **4a**, b, **4b** and c, **4c**). (Blue and red refer to a decrease and an increase in electron density, respectively)

Supplementary Table 1 Condition screening for the C-N coupling of **1** to aniline

Entry	Catalyst	Fold of aniline[a]	Phosphine ligand	Base	Yield
1	$Pd_2(dba)_3$	4	rac-BINAP[b]	Cs_2CO_3	31% (50%[c])
2	$Pd_2(dba)_3$	1	rac-BINAP	Cs_2CO_3	15%
3	$Pd_2(dba)_3$	1.5	rac-BINAP	Cs_2CO_3	18%
4	$Pd_2(dba)_3$	15	rac-BINAP	Cs_2CO_3	30%
5	$Pd_2(dba)_3$	30	rac-BINAP	Cs_2CO_3	31%
6	$Pd_2(dba)_3$	4	rac-BINAP	K_2CO_3	ND
7	$Pd_2(dba)_3$	4	rac-BINAP	tBu-OK	ND
8	$Pd_2(dba)_3$	4	Dppf[d]	Cs_2CO_3	13%
9	$Pd_2(dba)_3$	4	Xantphos[e]	Cs_2CO_3	15%
10	$Pd_2(dba)_3$	4	Bis(diphenylphosphino)methane	Cs_2CO_3	ND
11	$Pd_2(dba)_3$	4	1,2-Bis(diphenylphosphino)ethane	Cs_2CO_3	ND
12	$Pd_2(dba)_3$	4	X-phos[f]	Cs_2CO_3	trace[g]
13	$Pd_2(dba)_3$	4	Sphos[h]	Cs_2CO_3	trace[g]
14	$Pd_2(dba)_3$	4	BrettPhos[j]	Cs_2CO_3	ND
15	$Pd_2(dba)_3$	4	Pph_3	Cs_2CO_3	ND
16	$Pd_2(dba)_3$	4	$(t\text{-}Bu)_3P$	Cs_2CO_3	ND
17	$Pd_2(dba)_3$	4	Trihexylphosphine	Cs_2CO_3	ND
18	$Pd_2(dba)_3$	4	IMes•HCl[k]	Cs_2CO_3	ND

[a] according to the molar of chlorine at the vertexes of **1**

[b] (±)-2,2'-Bis(diphenylphosphino)-1,1'-binaphthalene;

[c] calculated by NMR spectroscopy

[d] 1,1'-Ferrocenebis(diphenylphosphine);

[e] 4,5-Bis(diphenylphosphino)-9,9-dimethylxanthene;

[f] 2-Dicyclohexylphosphino-2',4',6'-triisopropylbiphenyl; [g] detected by TLC

[h] 2-Dicyclohexylphosphino-2',6'-dimethoxybiphenyl;

[j] 2-(Dicyclohexylphosphino)-3,6-dimethoxy-2'-4'-6'-tri-i-propyl-1,1'-biphenyl;

[k] 1,3-Bis(2,4,6-trimethylphenyl)imidazolium chloride;

Supplementary Table 2 Absorption and photoluminescence data of **2a-2e**

	$\lambda_{max(abs)}$ (nm)[a] [ε(×10^4Lmol^{-1}cm^{-1})]	$\lambda_{max(em)}$ (nm)[a,b]	Stoke shift (cm^{-1})	PLQY (%)[c]	$E_{g,opt}$ (eV)[d]
**2a**	443 (6.3)	558	4652	3.14	2.18
**2b**	443 (7.3)	558	4652	3.55	2.18
**2c**	443 (9.0)	561	4748	5.45	2.15
**2d**	456 (10.6)	575	4539	-	2.10
**2e**	478 (14.0)	-	-	-	1.84

[a] in DCM. [b] excitation wavelength: 443 nm for **2a**; 443 nm for **2b**; 443 nm for **2c**; 456 nm for **2d.**

[c] Absolute photoluminescence quantum yield (PLQY) was determined by a calibrated integrating sphere. system. The PLQY of **2d** and **2e** is too low to be determined.

[d] Optical HOMO-LUMO gaps were estimated from the onset of the absorption spectra

Supplementary Table 3 Absorption data of **3**, **4a-4c**, **5**, **6a** and **6b**

	$\lambda_{max(abs)}$ (nm)[a] [ε(×10^4 cm^{-1} M^{-1})]	$E_{g,opt}$ (eV)[b]
**3**	446 (7.42)	2.11
**4a**	471 (13.9)	1.98
**4b**	473 (17.6)	1.95
**4c**	479 (14.0)	1.94
**5**	463 (null)	1.93
**6a**	500 (16.7)	1.72
**6b**	502 (14.0)	1.78

[a] in CH_2Cl_2. [b] Optical HOMO-LUMO gaps were estimated from the onset of the absorption spectra.

Supplementary Table 4 Comparison of theoretically calculated G and $G_{opt,\ exp.}$.

	$G_{opt,\ exp.}$ (eV)	G_{B3LYP} (eV)	$G_{CAM\text{-}B3LYP}$ (eV)	G_{M062X} (eV)	$G_{HSEH1PBE}$ (eV)
**2c**	**2.15**	2.73	4.91	4.51	**2.33**
**4b**	**1.95**	2.37	4.43	4.03	**1.96**

Supplementary Table 5 Comparison of the bond lengths (BL) between experimental data (crystal structure) and theoretically optimized structure of **2a** and relative deviations (RD) (See Supplementary Figure 51).

	C_1-$C_{1'}$ (Å)	C_1-C_2 (Å)	C_2-C_3 (Å)	C_3-C_3' (Å)	C_3-C_4 (Å)	C_4-C_5 (Å)	RD (%)[a]
Experimental	1.412	1.435	1.420	1.459	1.403	1.402	0
B3LYP	1.415	1.439	1.423	1.471	1.407	1.414	0.44%
CAM-B3LYP	1.406	1.442	1.413	1.470	1.400	1.406	0.44%
M062X	1.409	1.444	1.412	1.469	1.402	1.408	0.43%
HSEH1PBE	1.410	1.434	1.416	1.463	1.404	1.410	0.23%

[a] $RD = \Sigma[(|BL_{optimized} - BL_{experimental}|)/BL_{experimental}]/n$.
